# A comprehensive neuroimaging review of the primary and metastatic brain tumors treated with immunotherapy: current status, and the application of advanced imaging approaches and artificial intelligence

**DOI:** 10.3389/fimmu.2024.1496627

**Published:** 2024-11-28

**Authors:** Xiang Liu, Hongyan Chen, Guirong Tan, Lijuan Zhong, Haihui Jiang, Stephen M. Smith, Henry Z. Wang

**Affiliations:** ^1^ Department of Radiology, The Affiliated Yuebei People’s Hospital of Shantou University Medical College, Shaoguan, Guangdong, China; ^2^ Advanced Neuroimaging Laboratory, The Affiliated Yuebei People’s Hospital of Shantou University Medical College, Shaoguan, Guangdong, China; ^3^ Department of Radiology, Beijing Tiantan Hospital, Capital Medical University, Beijing, China; ^4^ Department of Pathology, The Affiliated Yuebei People’s Hospital of Shantou University Medical College, Shaoguan, Guangdong, China; ^5^ Department of Neurosurgery, Peking University Third Hospital, Peking University, Beijing, China; ^6^ Department of Imaging Sciences, University of Rochester Medical Center, Rochester, NY, United States

**Keywords:** immunotherapy, brain metastasis, malignant glioma, tumor progression, pseudoprogression, advanced imaging, MR perfusion imaging

## Abstract

Cancer immunotherapy has emerged as a novel clinical therapeutic option for a variety of solid tumors over the past decades. The application of immunotherapy in primary and metastatic brain tumors continues to grow despite limitations due to the physiological characteristics of the immune system within the central nervous system (CNS) and distinct pathological barriers of malignant brain tumors. The post-immunotherapy treatment imaging is more complex. In this review, we summarize the clinical application of immunotherapies in solid tumors beyond the CNS. We provide an overview of current immunotherapies used in brain tumors, including immune checkpoint inhibitors (ICIs), oncolytic viruses, vaccines, and CAR T-cell therapies. We focus on the imaging criteria for the assessment of treatment response to immunotherapy, and post-immunotherapy treatment imaging patterns. We discuss advanced imaging techniques in the evaluation of treatment response to immunotherapy in brain tumors. The imaging characteristics of immunotherapy treatment-related complications in CNS are described. Lastly, future imaging challenges in this field are explored.

## 1 Background

In the last few decades, clinical immunotherapy has provided exceptional achievements for many solid tumors. The clinical development of novel immunotherapies for the treatment of CNS tumors has also continued to grow. Compared to traditional tumor treatment (e.g. radiotherapy, chemotherapy, or surgery), the imaging evaluation of primary and metastatic brain tumors following immunotherapy treatment is much more challenging. To better understand post-immunotherapy treatment imaging characteristics in brain tumors, we provide an overview of the current clinical application of immunotherapies, with a focus on the advances of immunotherapeutic approaches employed in brain tumors. The imaging criteria for the assessment of treatment response to immunotherapy are summarized, with the highlight of post-immunotherapy treatment imaging patterns. The clinical experiences of advanced imaging techniques used for the evaluation of treatment response to immunotherapy in brain tumors are reviewed. The imaging characteristics of immunotherapy treatment-related complications in CNS are described. Lastly, we discuss future imaging challenges in this field.

## 2 Development of immunotherapy and clinical application in solid tumors

The development of immunotherapy has significantly advanced cancer treatment. As early as the 19th century, William B. Coley attempted the first cancer immunotherapy by injecting bacterial toxins into cancer patients (1). From the 1980s to the early 2000s, monoclonal antibody drugs were successively approved by the Food and Drug Administration (FDA) for preventing organ transplant rejection and treating B-cell non-Hodgkin lymphoma, marking the application of monoclonal antibodies in cancer treatment (2). In 2001, Brentuximab Vedotin was approved for Hodgkin lymphoma and systemic anaplastic large cell lymphoma (3). In 2010, the first therapeutic cancer vaccine, Sipuleucel-T, was approved by the FDA for prostate cancer, successfully utilizing the patient’s own immune cells for treatment (4).

**Figure 1:**
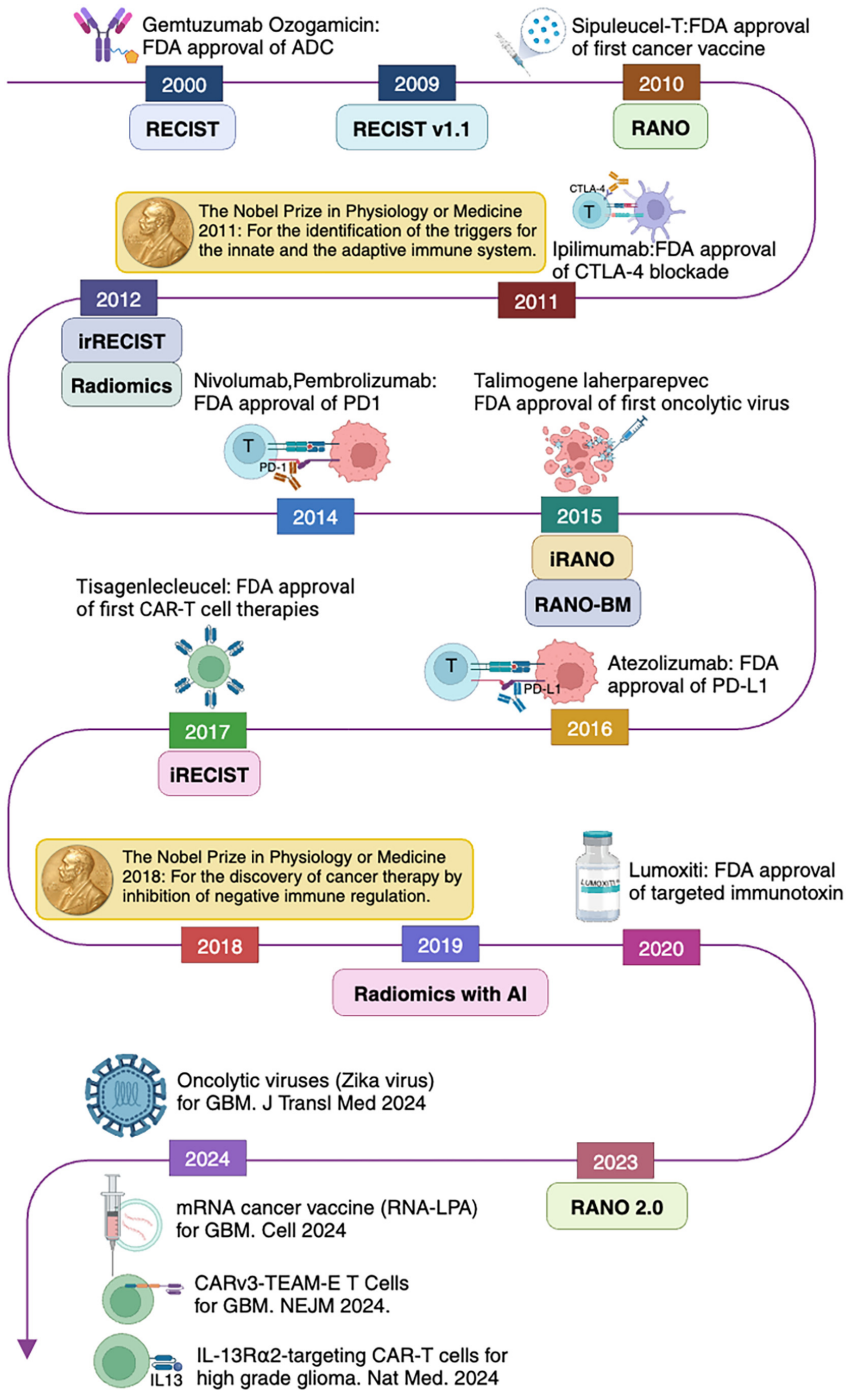
Evolution of cancer immunotherapy and imaging response assessment criteria related to brain tumors following immunotherapy.

In 2011, Ipilimumab was approved by the FDA for metastatic melanoma, becoming the first approved immune checkpoint inhibitor (ICI) (5). That same year, Ralph Steinman was awarded the Nobel Prize in Physiology or Medicine for his discovery of dendritic cells and their role in adaptive immunity. In 2014, programmed cell death protein 1 (PD-1) inhibitors, such as Pembrolizumab and Nivolumab, were approved for advanced melanoma, revolutionizing the field of cancer immunotherapy (6). In 2017, the FDA approved the first CAR-T cell therapy, Kymriah (Tisagenlecleucel), for acute lymphoblastic leukemia (ALL) and Yescarta (Axicabtagene Ciloleucel) for large B-cell lymphoma, marking a new era of personalized immunotherapy (3). In 2018, James P. Allison and Tasuku Honjo were awarded the Nobel Prize in Physiology or Medicine for their contributions to the development of ICIs **Figure 1**.

Currently, clinical immunotherapy primarily includes ICIs, cancer vaccines, oncolytic viruses, adoptive cell therapy (ACT), and cytokines **Table 1**.

**Table 1:** Summarization of immunotherapies in solid cancers beyond CNS.

Classification	Mechanism of Action	Representative Drugs	Applicable Cancer Types	Approval Status
Immune Checkpoint Inhibitors				
PD-1 Inhibitors	By blocking the binding of PD-1 to PD-L1, the immune function of T cells is restored, enhancing their ability to kill tumor cells	Pembrolizumab	Melanoma, NSCLC, RCC, TNBC, Gastric Cancer	FDA Approval
PD-1 Inhibitors		Nivolumab	Melanoma, NSCLC, RCC, Gastric Cancer	FDA Approval
PD-L1 Inhibitors		Atezolizumab	NSCLC, TNBC, Bladder Cancer	FDA Approval
PD-L1 Inhibitors		Avelumab	Bladder Cancer, Merkel Cell Carcinoma	FDA Approval
PD-L1 Inhibitors		Durvalumab	NSCLC, Bladder Cancer	FDA Approval
CTLA-4 Inhibitors	By blocking the binding of CTLA-4 to its ligands, T cell activation and anti-tumor immune responses are enhanced.	Ipilimumab	Melanoma, NSCLC, RCC	FDA Approval
LAG-3 Inhibitors	By blocking LAG-3 signaling, T cell proliferation and effector functions are enhanced, improving anti-tumor immune responses.	Relatlimab	Melanoma	In Clinical Trials
TIGIT Inhibitors	By blocking TIGIT signaling, the activity of T cells and NK cells is restored, enhancing their ability to kill tumor cells	Tiragolumab	SCLC	In Clinical Trials
IDO1 Inhibitors	By reducing immunosuppressive metabolic products, the effectiveness of PD-1 inhibitors is enhanced, boosting the anti-tumor activity of T cells.	Epacadostat	Melanoma	In Clinical Trials
Cancer Vaccine				
Peptide Vaccine	By injecting tumor-associated antigen peptide fragments, specific T cell responses are activated.	E75 (NeuVax), NY-ESO-1, IMA901	Breast Cancer, Melanoma, RCC, Ovarian Cancer.	Some vaccines are in the clinical trial stage.
DNA Vaccine	By injecting the DNA sequence encoding tumor antigens, cells can synthesize the antigens themselves and activate an immune response.	VGX-3100, INO-5401, RecombivaxHB, Engerix-B	Cervical cancer, prostate cancer, RCC, liver cance (HBV-related)	Some vaccines are in the clinical trial stage. Recombivax HB and Engerix-B have been approved by the FDA
Dendritic cell vaccine	Activating the patient’s immune system by loading dendritic cells with tumor antigens *in vitro*.	Sipuleucel-T (Provenge)	Prostate cancer	FDA Approval
mRNA vaccine	By introducing mRNA encoding tumor antigens into cells *in vivo*, these cells synthesize the tumor antigens and activate an immune response.	mRNA-4157, mRNA-5671	Melanoma, lung cancer, bladder cancer, pancreatic cancer, colorectal cancer.	Some vaccines are in the clinical trial stage.
Oncolytic virus vaccine	Genetically engineered viruses infect tumor cells, causing tumor cell lysis and death, and activate the immune system.	Talimogene laherparepvec (T-VEC)	Melanoma	FDA Approval
HPV vaccine	Inducing an immune response against HPV using virus-like particles (VLPs) to prevent HPV-related cancers.	Gardasil, Cervarix	Cervical cancer, anal cancer, head and neck cancer	FDA Approval
Oncolytic virus				
Based on direct cell lysis	By selectively infecting and lysing tumor cells	Talimogene laherparepvec (T-VEC)	Melanoma	FDA Approval
		Oncorine (H101)	Head and neck cancer	Approved in China in 2005
		Echovirus,Coxsackievirus	Melanoma	In Clinical Trials
Based on immune activation	Activating specific anti-tumor immune responses by inducing immunogenic cell death.	Reolysin (Pelareorep)	Head and neck cancer, breast cancer	In Clinical Trials
		Pexa-Vec (JX-594)	Hepatocellular carcinoma	In Clinical Trials
Based on the tumor microenvironment conditions	Modifying the tumor microenvironment to enhance immune cell activity	Measles Virus (MV)	Ovarian cancer	In Clinical Trials
Based on gene transfer	Enhancing the immune response by carrying and expressing anti-tumor genes.	Vaccinia Virus (VV)	Breast cancer, colorectal cancer	In Clinical Trials
		Adenovirus (AdV)	Prostate cancer	In Clinical Trials
Adoptive cell therapy				
Tumor-infiltrating lymphocytes (TIL)	Extracting and expanding naturally occurring tumor-infiltrating lymphocytes (TILs) within the tumor, then reinfusing them to enhance the anti-tumor immune response.	LN-144	Melanoma, cervical cancer, ovarian cancer.	In Clinical Trials
T-cell receptors (TCRs) that recognize specific cancer antigens (TCR)	Genetically engineering and introducing TCRs that recognize specific cancer antigens, then reinfusing them to enhance specific anti-tumor immune responses.	Kimmtrak (tebentafusp)	Metastatic or unresectable melanoma, synovial sarcoma	FDA Approval
Chimeric antigen receptor T cells (CAR-T)	Genetically engineering and introducing CARs that recognize specific antigens on the surface of cancer cells, then reinfusing them to enhance specific anti-tumor immune responses.	Kymriah (tisagenlecleucel)	Relapsed or refractory B-cell acute lymphoblastic leukemia (ALL) and large B-cell lymphoma	FDA Approval
		Yescarta (axicabtagene ciloleucel)	large B-cell lymphoma	FDA Approval
Cytokine therapy				
Interferon	Activating immune cells, enhance antigen presentation, inhibit tumor cell proliferation and angiogenesis	IFN-α	Melanoma, chronic myeloid leukemia, RCC	FDA Approval
Interleukin (ILs)	Promoting the proliferation and activation of T cells and natural killer cells, enhancing the immune system’s ability to attack cancer cells.	IL-2	Metastatic RCC, melanoma	FDA Approval
Tumor necrosis factor (TNFs)	To induce tumor cell apoptosis, disrupt tumor blood vessels, activate immune cells	TNF-α	Locally advanced soft tissue sarcoma and melanoma	FDA Approval

### 2.1 The application of immune checkpoint inhibitors in solid tumors (beyond the CNS)

ICIs have become standard treatments for various solid tumors beyond the CNS (7). PD-1 or programmed death ligand-1 (PD-L1) inhibitors primarily act on T cells within the tumor microenvironment, restoring their cytotoxic function by blocking the interaction between PD-1 and PD-L1. Representative drugs include PD-1 inhibitors nivolumab and pembrolizumab, as well as PD-L1 inhibitors atezolizumab, avelumab, and durvalumab, all of which have been approved by the FDA. These inhibitors are commonly used for treating malignant tumors such as melanoma, non-small cell lung cancer (NSCLC), renal cell carcinoma (RCC), triple-negative breast cancer (TNBC), gastric cancer, and gastroesophageal junction cancer (8. Cytotoxic T-Lymphocyte-Associated Protein 4 (CTLA-4) inhibitors act mainly during the early stages of T cell activation, blocking the negative regulatory effects of CTLA-4 that reduce T cell activation and proliferation. A representative drug is Ipilimumab, the first FDA-approved CTLA-4 inhibitor, indicated for melanoma and often used in combination with other ICIs for treating RCC and NSCLC (5).

PD-1/PD-L1 and CTLA-4 inhibitors have shown significant efficacy in cancer treatment but also face issues such as resistance, side effects, and limited response rates. Therefore, the other novel immune inhibitors that regulate immune responses through different mechanisms have emerged in recent years. These new inhibitors have the potential to address these shortcomings and demonstrate synergistic effects when used in combination with existing ICIs, further enhancing antitumor efficacy. For example, the combination of T cell immunoglobulin and mucin domain-containing protein 3 (TIM-3) inhibitors with PD-1/PD-L1 inhibitors has shown significant synergy in hepatocellular carcinoma (HCC) (9). T cell immunoreceptor with Ig and immunoreceptor tyrosine‐based inhibition motif domains (TIGIT) inhibitors combined with PD-1 inhibitors have been used to treat small cell lung cancer (SCLC) (10). Although the initial response to ICIs treatment is encouraging, many patients still can develop resistance after an initial response, resulting in the exploration of new combination therapy strategies and the identification of biomarkers to predict the efficacy of ICIs as a current research focus.

### 2.2 The application of cancer vaccines in solid tumors (beyond the CNS)

Cancer vaccines, by activating the patient’s immune system to recognize and attack tumor cells, have become a forefront area of immunotherapy research. The development of cancer vaccines aims to induce an immune response by introducing specific tumor antigens, thereby stimulating the body’s own immune protection mechanisms to achieve the treatment of tumors and/or the prevention of recurrence. The success of cancer vaccines requires the highly regulated cooperation of many branches of the immune system, including antigen-presenting cells, helper and cytotoxic T cells, natural killer (NK) cells, and tumor-resident myeloid cells (11).

The earliest cancer vaccines, peptide vaccines, emerged in the 1990s. By injecting tumor-associated antigen peptide fragments, these vaccines enhance the recognition and attack of tumor cells and are suitable for breast cancer, melanoma, RCC, and ovarian cancer (12). Subsequently, DNA vaccines appeared, activating T cells and B cells by injecting DNA encoding tumor antigens. These are commonly used to treat melanoma, prostate cancer, cervical cancer, and head and neck cancers (13).

In recent years, emerging dendritic cell vaccines and mRNA vaccines have made significant progress in the treatment of solid tumors. Dendritic cell vaccines activate T cells by loading them with tumor antigens (14). Sipuleucel-T, which appeared in 2010, is the first FDA-approved therapeutic cancer vaccine for hormone-refractory prostate cancer. mRNA vaccines induce immune responses by delivering mRNA encoding tumor antigens (4). Between 2018 and 2021, personalized mRNA cancer vaccines and vaccines combined with immune inhibitors were applied to melanoma, breast cancer, lung cancer, bladder cancer, pancreatic cancer, and colorectal cancer (15). Other vaccines, such as human papillomavirus (HPV) and hepatitis B virus (HBV) vaccines, have been approved by the FDA to prevent virus-related cervical cancer and liver cancer (16). With technological advances, the application of cancer vaccines in the treatment of solid tumors continues to grow.

### 2.3 The application of oncolytic viruses in solid tumors (beyond the CNS)

Oncolytic viruses achieve anticancer effects through selective infection and lysis of tumor cells. The molecular and cellular mechanisms are not yet fully understood but may involve several factors: direct cell lysis, induction of immunogenic cell death to activate specific antitumor immune responses, alteration of the tumor microenvironment to enhance immune cell activity, carrying antitumor genes to further boost effects of synergistic therapies, and disrupting the tumor vascular system to deprive tumor cells of nutrients and oxygen (17, 18). Over the past few decades, many different oncolytic virus candidates have been developed and tested (17). Most recently, scientists have combined oncolytic viruses with other current immunotherapies to turn “immune-cold” tumors into “immune-hot” tumors (19). These mechanisms make oncolytic viruses a promising cancer immunotherapy approach.

Oncolytic viruses have demonstrated the potential for various mechanisms in cancer treatment. By selectively infecting and lysing tumor cells, oncolytic viruses, such as T-VEC and Oncorine (H101), are currently used for the treatment of melanoma and head and neck cancers (20, 21). Immunoactivating viruses, such as Reolysin (Pelareorep) and Pexa-Vec (JX-594), are commonly applied to head and neck cancer, breast cancer, and HCC (22, 23). Viruses that modify the tumor microenvironment to enhance immune cell activity, such as the measles virus, are often used for melanoma and ovarian cancer (24). Viruses that carry and express antitumor genes to enhance immune responses, such as poxvirus and adenovirus, are used for breast cancer, colorectal cancer, and prostate cancer (25, 26).

### 2.4 The application of adoptive cell therapy in solid tumors (beyond the CNS)

ACT involves extracting a patient’s own immune cells, particularly T cells, expanding and genetically modifying them *in vitro*, and then reinfusing them back into the patient to enhance the anti-tumor immune response. ACT has shown significant clinical efficacy in treating various malignant tumors and has become a crucial area of research in current cancer immunotherapy. The three main forms of ACT include Tumor-Infiltrating Lymphocytes (TIL), T-cell receptors (TCR) targeting specific cancer antigens, and Chimeric Antigen Receptor T cells (CAR-T) (27).

TIL therapy involves extracting TIL with anti-tumor activity from the patient’s tumor tissues, expanding them *in vitro*, and reinfusing them into the patient to enhance the anti-tumor immune response. It is currently being tested in melanoma, cervical cancer, and ovarian cancer (28). Unlike TIL therapy, TCR therapy involves expanding and reinfusing T cells that have been genetically engineered to express specific antigen-recognizing TCRs, commonly used for melanoma and synovial sarcoma (29). Like TCR therapy, CAR-T therapy also reinfuses T cells. However, CAR-T involves introducing a chimeric antigen receptor that recognizes specific antigens on the surface of cancer cells into the patient’s T cells. CAR-T is commonly used for B-cell ALL, large B-cell lymphoma, and other hematological malignancies (30).

### 2.5 The application of cytokine therapy in solid tumors (beyond the CNS)

Cytokine therapy is a treatment method that regulates and enhances the immune system’s response against tumors through cytokines. Cytokines are small, secreted proteins released by various cells under stress, such as interferon-α, interferon-γ, interleukin-2 (IL-2), interleukin-15 (IL-15), interleukin-21 (IL-21), and interleukin-12 (IL-12) (31). They play powerful immunomodulatory roles in the body and are critical components of the tumor microenvironment, where they can either inhibit or promote tumor development.

Common cytokine therapies include interferons, interleukins, and tumor necrosis factor (TNF). Interferons work by activating immune cells (e.g. NK cells and macrophages), enhancing antigen presentation, and boosting the antiviral and antitumor activity of cells. They are commonly used for treating melanoma, chronic myelogenous leukemia, and RCC (32). Interleukins promote the proliferation and activation of T cells and NK cells, thereby enhancing the immune system’s ability to attack cancer cells. They are frequently used for metastatic RCC and melanoma (33, 34). Tumor necrosis factor (TNF) combats tumors through multiple mechanisms including tumor cell apoptosis, disrupting tumor vasculature, and activating immune cells. TNF is often used for locally advanced soft tissue sarcoma and melanoma (35, 36).

## 3 Immunotherapy for brain tumors

High grade glioma is the most common and aggressive primary malignant brain tumor of the CNS, and brain metastasis is the most common intracranial malignancy in adults (37). Recently, immunotherapy treatments were used in primary and secondary brain tumors and provided encouraging results (38, 39). However, their efficacy and outcomes are still low.

Immunotherapy for brain tumors faces many challenges (40–42). Protected by the blood-brain barrier (BBB), many immunotherapeutic drugs have difficulty entering the brain, resulting in insufficient concentrations in brain tumors and limited therapeutic efficacy (43, 44). Compared to brain tumors, organs and tissues where body tumors are located (such as the lungs, liver, and kidneys) do not have the physical impedance of the BBB, allowing drugs to reach the tumor site more easily and exert their therapeutic effects. Drugs can directly reach the tumor site through the bloodstream, penetrate the tumor microenvironment, and more effectively stimulate the immune system to attack tumor cells (45). Additionally, although recent studies have confirmed the existence of a glymphatic system and various antigen-presenting cells (such as microglia, macrophages, astrocytes, and dendritic cells) in the brain, brain tissue lacks the classical lymphatic drainage system and has a limited inflammatory response compared to other organs. As a result, the brain has been termed immune-privileged (46, 47).

Compared to other tumors, brain tumors have fewer TIL and immune effector cells. As a result, they exhibit characteristics of “cold tumors,” which significantly affect the efficacy of therapies such as immune checkpoint blockade (48). Additionally, the tumor microenvironment in brain tumors contains many immunosuppressive cells and molecules, creating a highly immunosuppressive microenvironment (31, 49, 50). This not only inhibits the activity of the immune system, but also promotes immune evasion by the tumor, and allows tumor cells to avoid attack by the immune system. Additionally, brain tumor cells exhibit immunoresistance, further weakening the effectiveness of immunotherapy (51). The heterogeneity of tumor cell populations and frequent genetic mutations within brain tumors leads to varied responses to treatment among different subpopulations of tumor cells, subsequently increasing the complexity of treatment, and making it difficult for targeted therapies to cover all tumor cells (52, 53). **Table 2**. Therefore, future research needs to focus on developing comprehensive treatment strategies that can overcome these immunosuppressive mechanisms and heterogeneity challenges to improve the efficacy of immunotherapy for brain tumors.

**Table 2:** Immuno-related with features and current status of immunotherapies in primary and metastatic brain tumors.

Tumor characteristics and Immunotherapies		Solid tumors beyond the CNS	Brain tumors	
			Primary brain tumor: glioma	Non-primary tumor: brain metastases (BM)
Immune related characteristics of tumors		No Blood-Brain Barrier (BBB). Body tumors, located in different organs, have rich lymphatic drainage system which facilitates the delivery of drugs and immune cells. Immune Environment: The immune environment of body tumors is relatively active, allowing immune cells to more easily recognize and attack tumor cells. However, different types of body tumors have different immune evasion mechanisms, which need to be specifically analyzed during treatment.	Glymphatic-lymphatic system in brain instead of lymphatic system. BBB is composed of tightly connected endothelial cells, and restrict the delivery of large molecule drugs and most immune cells to gliomas. Gliomas display increased BBB disruption as they progress. Immune Environment: The immune environment of the brain is relatively suppressive. The immunosuppressive microenvironment around gliomas makes it difficult for immune cells to infiltrate and function effectively. These cells include microglia and astrocytes, which play immunosuppressive roles in the tumor microenvironment.	Inefficient drug delivery due to the BBB/blood-tumor barrier (BTB) is a major dilemma in the systemic treatment of BM. Intact, lack of maturity or heterogeneously disrupted BBB or a lack of BBB can be observed in BM. BBB function in BM is tumor subtype specific (54, 55). Immune Environment: The immune response to brain metastases depends on the type of the primary tumor and its adaptation to the brain environment. Different types of metastatic tumors can exhibit significant variations in immune responses and physiological characteristics within the brain.
		Mutation Load: Body tumors typically have a high mutation load, making it easier for the immune system to recognize the aberrant tumor cells. Tumors with a high mutation load often have higher immunogenicity, capable of inducing a stronger immune response. Tumor Microenvironment: The microenvironment of body tumors contains a mix of immune cells that can either suppress or promote tumor growth, including tumor-associated macrophages, dendritic cells, and T cells. The interactions among these cells are complex. Body tumors are often “hot tumors”, they have high immune activity and are prone to eliciting an immune response.	Mutation Load: Gliomas have a relatively low mutation load, resulting in weaker immunogenicity. The low mutation load makes it more difficult for the immune system to recognize and attack tumor cells. Tumor Microenvironment: The microenvironment of gliomas is complex and contains various immunosuppressive cells. These cells not only inhibit immune responses but also promote tumor growth and invasion by secreting various factors. Tumor immune microenvironment of high grade gliomas is extremely complex and heterogeneous. The low mutation load and immunosuppressive microenvironment make gliomas form “cold tumors”.	Mutation Load: Different types of metastatic tumors may have significant differences in immunogenicity and mutation load within the brain. Tumor Microenvironment: The microenvironment of brain metastases depends on the type of primary tumor and the patient’s immune status. Primary tumors typically exhibit higher immune activity, making “hot tumors”.
Cancer Immunotherapy types	Immune checkpoint inhibitor			
	PD-1	FDA approval	Clinical trials	FDA approval for brain metastases from melanoma, lung and breast cancers
	PD-L1	FDA approval	Clinical trials	FDA approval for brain metastases from melanoma, lung and breast cancers
	CTLA-4	FDA approval	Clinical trials	FDA approval for brain metastases from melanoma
	Cancer Vaccine			
	Peptide Vaccine	Clinical trials	Not applicable	Clinical trials in brain metastases from melanoma
	DNA Vaccine	Clinical trials	Not applicable	Not applicable
	Dendritic cell vaccine	FDA approval	Clinical trials	FDA approval for brain metastases from prostate cancer
	mRNA vaccine	Clinical trials in melanoma, lung cancer, bladder cancer, pancreatic cancer and colorectal cancer	Not applicable	Not applicable
	Oncolytic virus vaccine	Clinical trials in melanoma	Clinical trials	Not applicable
	Oncolytic virus			
	Naturally Oncolytic Viruses	Clinical trials in melanoma, breast cancer and head and neck cancer	Clinical trials	Clinical trials
	Genetically Engineered Oncolytic Viruses	FDA approval	Clinical trials	Clinical trials in brain metastases from melanoma
	Adoptive cell therapy			
	TIL	FDA approval	Not applicable	Not applicable
	TCR	Clinical trials in metastatic or unresectable melanoma and synovial sarcoma	Not applicable	Not applicable
	CAR-T	FDA approval	Clinical trials	Clinical trials in brain metastases from melanoma
	Cytokine therapy			
	Interferon	FDA approval	Clinical trials	Clinical trials in brain metastases from melanoma
	Interleukin	FDA approval	Clinical trials	Clinical trials in brain metastases from melanoma
	Tumor necrosis factor	Clinical trials in locally advanced soft tissue sarcoma and melanoma	Clinical trials	Not applicable

We will separately introduce the immunotherapy of primary malignant gliomas and brain metastases (BM). By deeply analyzing the immunotherapy of these specific types of brain tumors, **Table 2**. We hope to provide valuable insights and guidance for future research and clinical practice.

### 3.1 Immunotherapy for gliomas

Glioblastoma multiforme (GBM) is the most common type of primary malignant brain tumor in adults (56). The current standard of care, proposed by Stupp et al. in 2005, includes maximal surgical resection followed by radiotherapy and concurrent chemotherapy with temozolomide (57). This regimen has been widely accepted and is the global standard for treating GBM. Despite this multimodal approach, the prognosis for patients remains poor, with a median overall survival of approximately 14-16 months (58). In recent years, immunotherapy has significantly improved outcomes in many systemic tumors, bringing new hope for improved treatment of high-grade gliomas (59). Here are some of the main immunotherapy approaches applied to gliomas.

#### 3.1.1 The application of immune checkpoint inhibitors in gliomas

Research on ICIs for gliomas has primarily focused on the PD-1/PD-L1 and CTLA-4 pathways. In gliomas, the use of common PD-1 inhibitors, such as nivolumab and pembrolizumab, faces several challenges, including the limitations of the BBB and the complexity of tumor immune evasion mechanisms. However, some clinical trials have shown certain efficacy (59–61). For example, trials of nivolumab in patients with recurrent GBM have indicated that some patients respond to the treatment and have extended survival (62). The common CTLA-4 inhibitor ipilimumab has also been shown to be effective for the treatment of recurrent GBM. Although its effectiveness as a monotherapy is limited, combining it with other treatments may enhance outcomes (62). Currently, there are multiple ongoing research on the combination of PD-1 and CTLA-4 inhibitors, with the hope of improving overall response rates in patients (63).

Targeted therapy, via inhibition of specific cancer-related signaling pathways such as EGFR and VEGF, has shown significant efficacy in various tumors (64–66). Combining targeted therapy with ICIs is expected to enhance treatment effects through multiple mechanisms (67). Several clinical studies are exploring the combined application of ICIs and targeted therapies in GBM. For example, the combination of the VEGF-targeted drug bevacizumab with a PD-1 inhibitor can reduce tumor angiogenesis and improve immune cell infiltration into tumors.

#### 3.1.2 The application of cancer vaccines in gliomas

Cancer vaccines are designed to induce the immune system to recognize and attack specific tumor antigens (13, 68). Research on vaccines for gliomas has primarily focused on the EGFRvIII vaccine and dendritic cell vaccines (69–71). EGFRvIII is a mutation commonly found in GBM. Vaccines developed against EGFRvIII, such as Rindopepimut, have shown promising immune responses in clinical trials (72). Although it did not significantly extend patient survival in Phase III clinical trials, research on this vaccine continues. Dendritic cell vaccines for gliomas have also shown some immune responses and extended survival in initial clinical trials, but their efficacy still needs further validation (73). In 2024, novel mRNA cancer vaccines applied to brain tumors have significantly improved treatment effects by activating anti-tumor immune responses (74). Additionally, the attenuated Zika virus vaccine (ZIKV-LAV) has shown potential therapeutic effects against human GBM (75).

#### 3.1.3 The application of oncolytic viruses in gliomas

In the research on immunotherapy for gliomas, oncolytic virotherapy has shown significant potential and emerged as a promising treatment strategy (76–78). Talimogene laherparepvec (T-VEC), a genetically modified herpes simplex virus, has achieved success in melanoma and is now being studied for gliomas (79). It can directly kill tumor cells and elicit a strong immune response by releasing tumor antigens and cytokines. Additionally, the modified adenovirus DNX-2401 has demonstrated selective killing of GBM cells in clinical trials, significantly reducing tumor size and achieving long-term survival in some patients (80, 81).

Toca 511, a non-lytic amphotropic retroviral replicating vector, has shown encouraging efficacy in early studies of recurrent high-grade gliomas, with some patients achieving complete remission (82). G207, a genetically modified type I oncolytic herpes simplex virus, has been shown to significantly increase the number of TIL in gliomas and successfully convert “cold” tumors into “hot” tumors, paving the way for new directions in glioma immunotherapy (83, 84). New research indicates that CAN-3110, another oncolytic virus derived from herpes simplex virus, has also demonstrated good immune activation effects and potential therapeutic benefits in clinical trials (85). Overall, the application of oncolytic virotherapy in gliomas is advancing and may provide effective complementation to traditional treatments and potential efficacy improvements.

#### 3.1.4 The application of adoptive cell therapy in gliomas

The application of ACT in gliomas shows promising prospects, particularly in the innovative research on CAR-T cell therapy, TIL therapy, TCR-T cell therapy, NK cell therapy, and CAR-NK cell therapy (86–89). CAR-T cell therapy has achieved remarkable success in hematologic malignancies, but its application in gliomas is still under exploration. CAR-T cell therapies targeting EGFRvIII and IL-13Rα2 are currently in clinical trials. In a recent phase I clinical trial for recurrent high-grade gliomas, local administration of IL-13Rα2-targeted CAR-T cells demonstrated safety and preliminary efficacy, with some patients achieving stable disease (SD) or even remission (90). However, due to the heterogeneity and immune evasion mechanisms of gliomas, the durability and overall efficacy of this therapy require further investigation.

TIL therapy has shown efficacy in melanoma, but research in gliomas is still in the early stages. Some preliminary studies indicate its potential to induce anti-tumor immune responses (28, 91). Like CAR-T cell therapy, TCR-T cell therapy faces challenges such as the BBB, tumor heterogeneity, and immune evasion. Clinical trials for TCR-T cell therapy targeting gliomas are ongoing (92).

NK cell therapy has shown some anti-tumor potential in gliomas, especially when combined with other immunotherapy methods (93). CAR-NK (Chimeric Antigen Receptor-Natural Killer) cell therapy applies CAR technology to NK cells, giving them the ability to specifically recognize and attack tumor cells. The application of CAR-NK cell therapy in gliomas is still in the early research stages, but preliminary results show promise (94).

#### 3.1.5 The application of cytokine therapy in gliomas

Common cytokine therapies include IL-2, interferon-γ (IFN-γ), IL-12, TNF-α, and IL-15 (95). Due to the strong immune activation effects of cytokines, they bring potential toxicity and side effects. As a result, the systemic use of cytokine therapy in the treatment of GBM is limited. Currently, the application of cytokine therapy to GBM is still in the research stage (96, 97).

### 3.2 Immunotherapy for brain metastases

BM is the most common intracranial malignancy in adult cancer patients, with an estimated 30-40% of patients with solid malignancies developing intracranial metastases during their illness (98). Over the past decade, the incidence of BM has increased. As the population ageing and the number of cancer diagnoses markedly increase, the likelihood of BM also increases. Additionally, improvements in systemic treatments have extended the survival time of cancer patients, leading to a projected continued increase in the incidence of BM (99). Common primary cancers that metastasize to the brain include lung cancer, breast cancer, melanoma, and kidney cancer (100).

Historically, the diagnosis of BM has been considered fatal, almost akin to a death sentence, with only a few treatment options available to alleviate symptoms or extend life (100). Among the limited treatment options, brain radiation therapy and surgical resection have been the mainstays of treatment (100). In recent years, immunotherapy, either used alone or in combination with traditional treatments, has emerged as a significant force in combating the spread of BM and reducing tumor burden (100). Next, we will introduce the application of immunotherapy in the treatment of BM from various types of cancer.

#### 3.2.1 Application of immune checkpoint inhibitors in brain metastases

ICIs have shown significant efficacy in the treatment of BM from various types of cancer (101). In recent years, ICIs such as ipilimumab, pembrolizumab, and nivolumab have been increasingly used in patients with melanoma BM (102–104). In the application for lung cancer, studies have found that PD-1/PD-L1 inhibitors as monotherapy have demonstrated good results in advanced NSCLC BM patients (105). Some case reports have shown that patients with SCLC BM experienced a complete disappearance of recurrent BM and sustained complete remission after receiving a combination of multi-kinase inhibitors and PD-1/PD-L1 inhibitors (106).

Compared to melanoma and lung cancer, the application of immune inhibitors in breast cancer BM is less developed. Some recent studies have found that combining anti-PD-1 therapy with anti-estrogen therapy can significantly extend progression-free survival in patients, although results still require more research for validation (107). Colorectal cancer and RCC have a relatively lower incidence of BM (108, 109), but immunotherapy has also shown some promise in these cancer types. For example, the use of nivolumab in patients with RCC BM has shown good safety and efficacy (110).

#### 3.2.2 The application of cancer vaccines in brain metastases

Dendritic cell vaccines are an important direction in cancer vaccine research. Studies have found that these vaccines can significantly enhance tumor antigen-specific T-cell responses and prolong overall survival in patients with BM (111). Combining dendritic cell vaccines with ICIs is another strategy to improve therapeutic outcomes. For example, using an anti-HER2/HER3 dendritic cell vaccine in combination with Pembrolizumab (a PD-1 inhibitor) can significantly improve CNS response rates and progression-free survival in patients with HER2-positive breast cancer BM (112). A study showed that a patient with colorectal cancer BM who received a dendritic cell vaccine pulsed with tumor antigens exhibited a significant antitumor response (113). Despite the promising effects of cancer vaccines in treating BM, further research is needed to optimize vaccine design and treatment strategies.

#### 3.2.3 The application of oncolytic viruses in brain metastases

In the treatment of BM, research on oncolytic viruses has primarily focused on several specific viruses, such as adenovirus, herpes simplex virus, and measles virus (114). T-VEC, an oncolytic virus based on HSV-1, has been approved for the treatment of advanced melanoma and has shown potential in treating BM (115). In combination with other treatment modalities, oncolytic viruses have also demonstrated significant synergistic effects. For example, the combination of oncolytic viruses with ICIs has been used to treat unresectable melanoma and has shown promising therapeutic outcomes in cases involving BM (116, 117). Research indicates that oncolytic viruses can enhance the efficacy of ICIs by lysing tumor cells and releasing tumor antigens. Additionally, the combination of oncolytic viruses with radiotherapy has shown promising results (118) as radiotherapy can increase the immunogenicity of tumor cells, thereby enhancing the antitumor effects of oncolytic viruses (115).

#### 3.2.4 Adoptive cell therapy in the treatment of brain metastases

The application of ACT in BM primarily focuses on several common types of BM. TILs therapy has shown significant efficacy in melanoma BM. In one study, a notable proportion of patients receiving TILs therapy exhibited tumor reduction and prolonged survival (119). CAR-T cell therapy has shown potential in treating breast cancer BM and CAR-T cell therapy targeting HER2-positive breast cancer is currently undergoing clinical trials (120). The application of ACT in NSCLC BM is less studied, but preliminary research suggests that TCR-T cell therapy has the potential to specifically recognize and kill tumor cells (121).

#### 3.2.5 The application of cytokine therapy in brain metastases

IL-2 and IL-12 are among the cytokines most extensively studied in the treatment of BM. Benefiting from the similarity between BM and primary body tumors, some cytokine therapies that have shown significant efficacy in melanoma also demonstrate promising performance in the treatment of BM. Studies have shown that the combination therapy of IFN-α and IL-2 can significantly extend the progression-free survival (PFS) and overall survival (OS) of some melanoma patients with BM (122).

### 3.3 Immunotherapy for primary central nervous system lymphoma

Primary central nervous system lymphoma (PCNSL) is a rare and aggressive form of non-Hodgkin lymphoma that primarily affects the lymphatic tissue in the brain, spinal cord, and eyes (123). Chemotherapy and radiotherapy are the main treatment modalities for PCNSL. Although traditional chemotherapy and radiotherapy can be effective, the recurrence rate of PCNSL remains high (124). In recent years, immunotherapy has shown great promise in the treatment of primary central nervous system lymphoma, especially with the advent of CAR T-cell therapy and ICIs (125, 126). While these methods are currently in the clinical research phase, they hold the potential to bring new treatment options to PCNSL patients, potentially extending survival and improving quality of life.

### 3.4 Immunotherapy for meningioma

Meningioma is the most common type of primary intracranial tumor, with the majority being grade I, which can often be cured with complete surgical resection. However, grade II and grade III meningiomas frequently recur after surgery and radiotherapy, making their treatment more challenging (127). Traditional alkylating agent chemotherapy and targeted therapies are generally ineffective against these high-grade meningiomas.

In recent years, immunotherapy for high-grade meningiomas has garnered widespread attention. Research has found that the expression of immune checkpoint molecules, such as PD-L1, in meningiomas is positively correlated with tumor grade. This suggests that high-grade meningiomas may be more responsive to treatment with ICIs (128, 129). A phase II trial using nivolumab for patients with recurrent grade II and III meningiomas showed an improvement in median progression-free survival compared to previous studies with other therapies (127). With the advancement of immunotherapy, future research should focus on gaining a better understanding of the impact of various cellular components within the tumor microenvironment of meningiomas on treatment outcomes, in order to further enhance the precision and effectiveness of these therapies.

### 3.5 Summary

The application of immunotherapy in brain tumors, particularly in primary brain tumors and BM, demonstrates tremendous potential. By further understanding the immune microenvironment of brain tumors, overcoming the obstacles posed by the BBB, and developing new immunotherapeutic approaches, immunotherapy is poised to become a significant modality in the treatment of brain tumors.

## 4 Evolution of cancer imaging evaluation criteria

Post-treatment cancer imaging is critically important for clinical management in oncology practice. For better treatment response evaluation, multiple cancer imaging evaluation strategies have been proposed, including World Health Organization criteria (130), which were introduced in 1979, and RECIST 1.0, which were introduced in 2000 (131) and revised (RECIST 1.1) in 2009 (132), **Figure 1**. The first set of imaging criteria for evaluating immunotherapy treatment was the immune-related response criteria, which were developed in 2009 (133). Further modifications were attempted to accurately characterize immune-related response patterns. These efforts resulted in modified criteria including immune-related RECIST (irRECIST) which was developed in 2013 (134), and iRECIST in 2017 (135).

**Figure 2:**
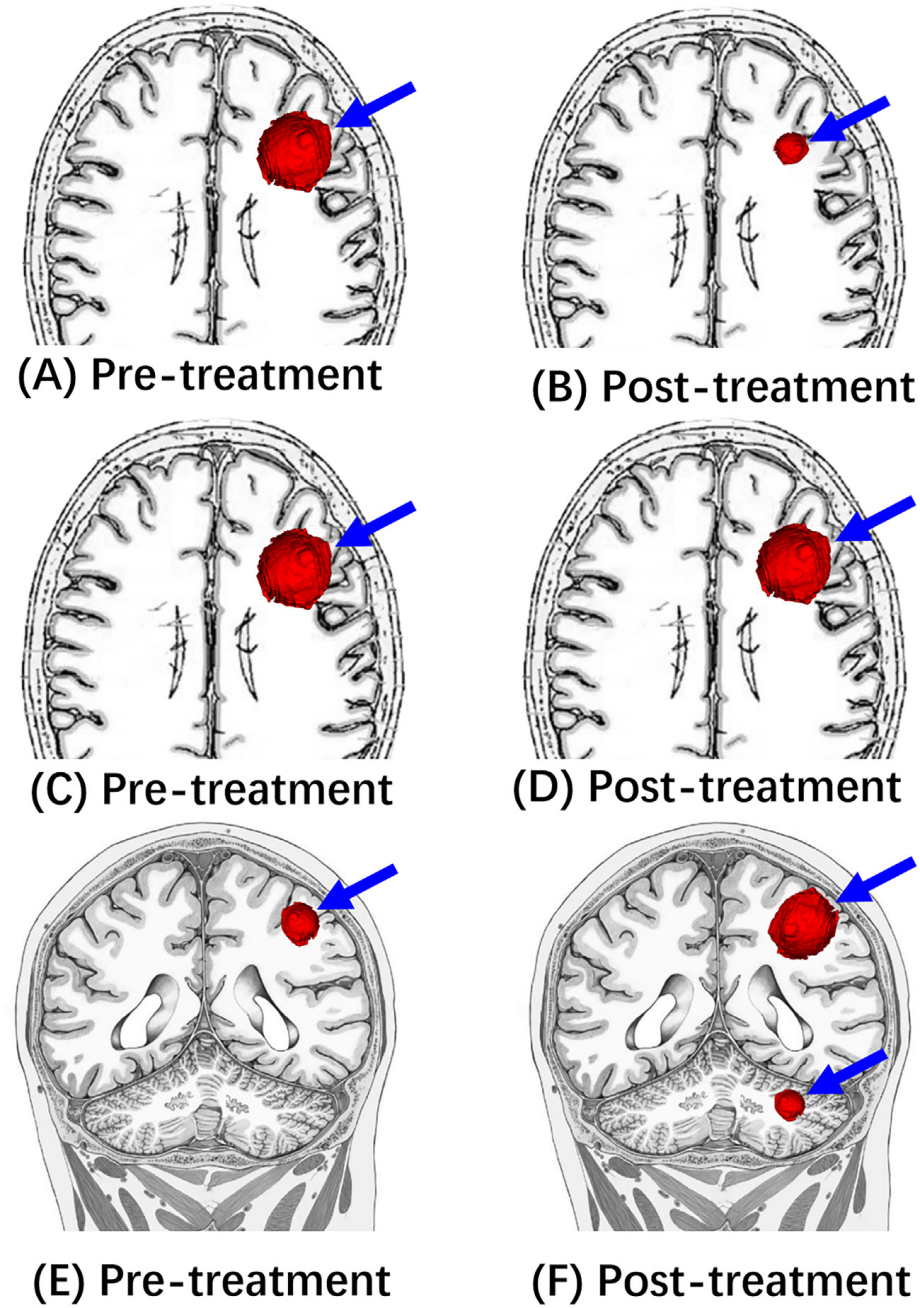
Illustrative diagrams of Response (PR), Stable (SD), and Progression (PD). **(A, C, E)** Are pre-treatment MRI showing a left frontal mass (blue arrow). After the immunotherapy treatment, **(B)** shows the decreased size of this mass (blue arrow), which is consistent with “Response”(PR). **(D)** Shows “stable” (SD) tumor size following the immunotherapy treatment. **(F)** Shows “progression” (PD) of tumor size following the immunotherapy treatment.

**Figure 3:**
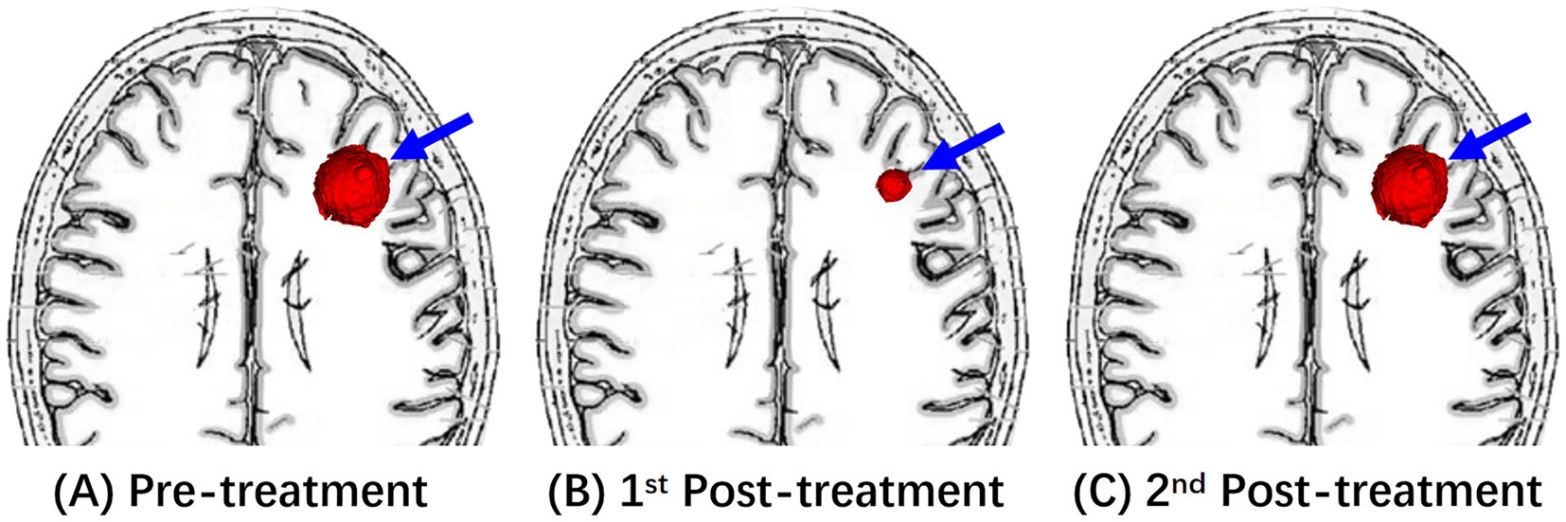
An illustrative diagram of Response (PR). **(A)** Shows a left frontal mass on pre-treatment MRI (blue arrow). After the immunotherapy treatment, **(B)** of the 1st post-treatment MRI showed the decreased size of this mass. The 2nd post-treatment MRI of **(C)** reveals re-enlargement of this enhancing mass (blue arrow).

In the field of neuro-oncology, the Response Assessment in Neuro-Oncology (RANO) Working Group published a series of response criteria for high-grade gliomas (RANO-HGG) in 2010 (136), low-grade gliomas (RANO-LGG) in 2011 (137), the Immunotherapy RANO Criteria (iRANO) (138), and the RANO Criteria for BM (RANO-BM) (139) in 2015, and the Modified RANO Criteria (mRANO) in 2017 (140). The latest updated RANO criteria were RANO 2.0 which includes both high-grade and low-grade gliomas, were published in 2023 (141). The iRANO, RANO-BM, and RANO 2.0 are three RANO criteria related to immunotherapy treatment response evaluation for malignant glioma and BM. We compare these criteria, including brain tumor types, definitions of the measurement methods, imaging evaluation baseline, targets, and response criteria including complete response (CR) (203), partial response (PR), SD, and progressive disease (PD) in **Table 3**, **Figures 2** and **3**.

**Table 3:** Comparison of RANO2.0, iRANO, RANO-BM criteria in the response of brain tumors treated with immunotherapy.

Imaging Assessment Criteria	RANO 2.0	iRANO	RANO-BM
Reference	RANO 2.0: Update to the Response Assessment in Neuro-Oncology Criteria for High- and Low-Grade Gliomas in Adults. J Clin Oncol.2023 (141).	Immunotherapy Response Assessment in Neuro-Oncology (iRANO): A Report of the RANO Working Group. Lancet Oncol. 2015 (138).	Response assessment criteria for brain metastases: proposal from the RANO group. Lancet Oncol. 2015 (139).
Brain tumor types	glioblastomas, all grades of IDH - mutated gliomas, and other glial tumors	malignant glioma, low-grade glioma, and brain metastases	parenchymal brain metastases only and do not cover leptomeningeal metastases
Measurement	Two-dimensional tumor measurement will remain the recommended primary measurement, but volumetric measurements can be used if available.	Two-dimensional tumor measurement for malignant glioma and low-grade glioma. Unidimensional measurements (the longest diameter for brain metastases).	Unidimensional measurements (the longest diameter for solid component), similar to RECIST 1.1.
Measurable disease	Measurable disease is defined as contrast-enhancing or non-contrast-enhancing lesions with clearly defined margins by MRI scan, with both perpendicular diameters on a single slice of at least 10 mm, visible on two or more slices that are preferably, at most, 4 mm apart with 0-mm interslice gap. Volumetrically, measurable disease in 3D will be defined as having contrast-enhancing or non-enhancing-disease of at least 1 cm in all three orthogonal directions. For MRI performed with thicker slices, the size of a measurable lesion at baseline for both perpendicular measurements should be two times the slice thickness and interslice gap (eg, if the slice thickness is 5 mm with 1.5-mm interslice gap, the minimum tumor size on both perpendicular dimensions should be 13 mm).	Measurable disease is defined as bidimensionally contrast-enhancing lesions with clearly defined margins by CT or MRI scan, with two perpendicular diameters of at least 10 mm, visible on two or more axial slices that are preferably, at most, 5 mm apart with 0 mm skip.	Measurable disease is defined as a contrast-enhancing lesion that can be accurately measured in at least one dimension, with a minimum size of 10 mm, and is visible on two or more axial slices that are preferably 5 mm or less apart with 0 mm skip (and ideally ≤1.5 mm apart with 0 mm skip). Additionally, although the longest diameter in the plane of measurement is to be recorded, the diameter perpendicular to the longest diameter in the plane of measurement should be at least 5 mm for the lesion to be considered measurable. If the MRI is performed with thicker slices, the size of the measurable lesion at baseline should be at least double the slice thickness. Interslice gaps, if present, should also be considered in the determination of the minimum size of measurable lesions at baseline.
Non-measurable disease	Nonmeasurable disease remains defined as either unidimensionally measurable lesions, masses with unclear margins, or lesions with maximal perpendicular diameters <10 mm. Measurement of tumor around a cyst or surgical cavity remains challenging. Such lesions should generally be considered nonmeasurable unless there is a nodular component measuring ≥10 × 10 mm in diameter. The cystic or surgical cavity should not be measured in determining therapeutic response.	Nonmeasurable disease is defined as either unidimensionally measurable lesions, masses with margins not clearly defined, or lesions with maximal perpendicular diameters less than 10 mm. Measurement of tumor around a cyst or surgical cavity represents a particularly difficult challenge. In general, such lesions should be considered nonmeasurable unless there is a nodular component measuring 10 mm in diameter. The cystic or surgical cavity should not be measured in determining response.	Non-measurable disease includes all other lesions, including lesions with longest dimension less than 10 mm, lesions with borders that cannot be reproducibly measured, dural metastases, bony skull metastases, cystic-only lesions, and leptomeningeal disease. Measurement of a tumour around a cyst or surgical cavity is a particularly difficult challenge. Generally, such lesions should be considered non-measurable unless there is a nodular component that measures 10 mm or more in longest diameter and 5 mm or more in the perpendicular plane. The cystic or surgical cavity should not be measured for the determination of a response.
Baseline	The postradiotherapy MRI scan, performed around 4 weeks (21-35 days) from the end of radiotherapy, is recommend as the baseline scan in newly diagnosed gliomas for comparison with future imaging studies. For patients with newly diagnosed glioma not undergoing radiotherapy, the postsurgery, pretreatment MRI will be used as the baseline. The pretreatment MRI will also be used for patients with recurrent glioma as the baseline. Ideally, baseline scans should be performed as close as possible to the initiation of therapy with an interval not exceeding 14 days, especially for glioblastomas.	A baseline MRI scan for newly diagnosed glioma should ideally be obtained within 24 to 48 hours after surgery and no later than 72 hours after surgery, which was defined in RANO 2.0 (141). A baseline MRI scan for BM should be done as close as possible to the treatment start and no more than 4 weeks before the beginning of treatment (same as RANO-BM).	All baseline assessments should be done as close as possible to the treatment start and no more than 4 weeks before the beginning of treatment.
Target	When multiple measurable lesions exist, at least two and no more than three lesions should be identified as target lesions for studies evaluating either enhancing or nonenhancing tumors. For studies evaluating both enhancing and nonenhancing tumors, a maximum of two measurable enhancing and two measurable nonenhancing lesions can be identified as target lesions. The enhancing lesion(s) can be in the nonenhancing tumor. The sum of the products of the perpendicular diameters of these lesions should be determined. Occasionally, the largest lesions may not lend themselves to reproducible measurements, and the next largest lesions that can be measured reproducibly should be selected. For patients with multiple lesions, those that are increasing in size should be selected as target lesions, regardless of their relative size. The other lesions will be considered nontarget and should be recorded but not integrated into the total lesion size calculation.	If there are multiple contrast-enhancing lesions, a minimum of the two largest lesions should be measured, and the sum of the products of the perpendicular diameters of these lesions should be determined. In some lesions of high-grade gliomas, a maximum of five of the largest lesions may be measured. In general, the largest enlarging lesion(s) should be selected. Occasionally, the largest lesions may not lend themselves to reproducible measurements, and the next largest lesions that can be measured reproducibly should be selected. For patients with recurrent disease who have multiple lesions of which only one or two are increasing in size, the enlarging lesions should be considered the target lesions for evaluation of response. The other lesions will be considered nontarget lesions and should also be recorded. Rarely, unequivocal progression of a nontarget lesion requiring discontinuation of therapy or development of a new contrast-enhancing lesion may occur, even in the setting of stable disease or partial response in the target lesions. These changes would qualify as progression.	When more than one measurable lesion in the CNS is present at baseline, all lesions up to a maximum of five CNS lesions should be identified as target lesions and will be recorded and measured at baseline. All measurements should be recorded in metric notation. Target lesions should be selected on the basis of their size (longest diameter) and as those that can be measured reproducibly. For patients with recurrent disease who have multiple lesions, of which only one or two are increasing in size, the enlarging lesions should be prioritised as target lesions for the response assessment. Lesions with prior local treatment (ie, stereotactic radiosurgery or surgical resection) can be considered measurable if progression has occurred since the time of local treatment. If lesions not previously treated with local therapies are present, these are preferred for selection as target lesions. A sum of the diameters for all target lesions will be calculated and reported as the baseline sum of longest diameters. All other CNS lesions should be identified as non-target lesions and should also be recorded at baseline.
Complete response (CR)	Complete Response (CR) - *compare to baseline* disappearance of target and non-target lesions sustained for ≥4 weeks ^¶^ and no new lesions ^*^ and clinical status is stable or improved and off corticosteroids (or on physiologic replacement dose) (203)	Malignant Glioma: Disappearance of all enhancing disease for ≥4 weeks; no new lesions; stable or improved T2/FLAIR; no more than physiological steroids; clinically stable or improved. Low-Grade Glioma: Disappearance of all enhancing and T2/FLAIR disease for ≥4 weeks; no new lesions; no more than physiological steroids; clinically stable or improved. Brain Metastases: Disappearance of all enhancing target and non-target lesions for ≥4 weeks; no new lesions; no steroids; clinically stable or improved.	Target lesions Complete response Disappearance of all CNS target lesions sustained for at least 4 weeks; with no new lesions, no use of corticosteroids, and patient is stable or improved clinically. Non-target lesions Requires all of the following: disappearance of all enhancing CNS non-target lesions, no new CNS lesions.
Partial response (PR)	Partial Response (PR) – *compare to baseline* at least 50% decrease in tumor burden with 2D measurements,or 65% with 3D measurements, sustained for ≥4 weeks ^† ¶^and no newly-measurable lesions ^* Δ °^ and clinical status is stable or improved and corticosteroid dose is stable compared to baseline (or on physiologic replacement dose) (203).	Malignant Glioma: ≥50% decrease in the sum of biperpendicular diameters of enhancing disease for ≥4 weeks; no new lesions; stable or improved T2/FLAIR; stable or decreased steroid dose; clinically stable or improved. Low-Grade Glioma: ≥50% decrease in the sum of biperpendicular diameter of T2/FLAIR disease for ≥4 weeks; no new lesions; stable or decreased steroid dose; clinically stable or improved. Brain Metastases: ≥30% decrease in sum of longest diameters of target lesions for ≥4 weeks; no new lesions; stable or decreased steroid dose; clinically stable or improved.	Target lesions: At least a 30% decrease in the sum longest diameter of CNS target lesions, taking as reference the baseline sum longest diameter sustained for at least 4 weeks; no new lesions; stable to decreased corticosteroid dose; stable or improved clinically. Non-target lesions: Non-complete response or non-progressive disease Persistence of one or more non-target CNS lesion or lesions.
Minor response (MR) applies only to nonenhancing disease	Minor Response (MR, only applicable to non-CE disease)– *compare to baseline* 25% to 50% decrease in tumor burden with 2D measurements, or 40% to 65% with 3D measurements, sustained for ≥4 weeks ^¶^ and no newly-measurable lesions ^* Δ °^ and clinical status is stable or improved and corticosteroid dose is stable compared to baseline (or on physiologic replacement) (203).	Malignant Glioma: Non applicable. Low-Grade Glioma: 25–49% decrease in the sum of biperpendicular diameters of T2/FLAIR disease for ≥4 weeks; no new lesions; clinically stable or improved. Brain Metastases: Not applicable.	Not applicable
Stable disease (SD)	Stable Disease (SD) all scenarios that do not meet criteria for CR, PR, MR, or PD.e.g., stable radiographic findings without clinical deterioration. (203)	Malignant Glioma Does not qualify for complete response, partial response, or progressive disease; no new lesions; stable or improved T2/FLAIR; stable or decreased steroid dose; clinically stable or improved. Low-Grade Glioma: Does not qualify for complete response, partial response, or progressive disease; no new lesions; stable or improved T2/FLAIR; stable or decreased steroid dose; clinically stable or improved. Brain Metastases: Does not qualify for complete response, partial response, or progressive disease	Target lesions Neither sufficient shrinkage to qualify for partial response nor sufficient increase to qualify for progressive disease, taking as reference the smallest sum longest diameter while on study. Non-target lesions Non-complete response or non-progressive disease Persistence of one or more non-target CNS lesion or lesions.
Progressive disease (PD)	Progressive Disease (PD) ^‡^ – *compare to nadir* at least 25% increase in tumor burden with 2D measurements, or 40% with 3D measurements, with or without confirmation scan after ≥4 weeks ^§^ or appearance of newly-measurable lesions ^* Δ °^ or appearance of leptomeningeal disease or clinical deterioration not ascribable to steroid dose reduction or other causes apart from the tumor or failure to return for evaluation as a result of death or clinical deterioration (203)	Malignant Glioma: ≥25% increase in the sum of biperpendicular diameters of enhancing disease; or new lesions; or substantial worsened T2/FLAIR; or substantial clinical decline. Low-Grade Glioma: ≥25% increase in the sum of biperpendicular diameters of T2/FLAIR disease; or new lesions; or substantial clinical decline. Brain Metastases: ≥20% increase in the sum of longest diameters of target lesions; or unequivocal progression of enhancing non-target lesions; or new lesions; or substantial clinical decline 1. No new or significantly worsened neurologic deficits not due to co-morbid event or concurrent medication AND 2. ≤ 6 months from initiation of immunotherapy. If follow-up imaging confirms progression, the date of actual progression should be back-dated to the date of initial radiographic progression. • Appearance of new lesions solely does not define progressive disease≤ 6 months from initiation of immunotherapy. The lesions are added to the total lesion areas for follow-up.	Target lesions At least a 20% increase in the sum longest diameter of CNS target lesions, taking as reference the smallest sum on study (this includes the baseline sum if that is the smallest on study). In addition to the relative increase of 20%, at least one lesion must increase by an absolute value of 5 mm or more to be considered progression. Non-target lesions Any of the following: unequivocal progression of existing enhancing non-target CNS lesions, new lesion(s) (except while on immunotherapy-based treatment), or unequivocal progression of existing tumour-related non-enhancing (T2/FLAIR) CNS lesions. In the case of immunotherapy-based treatment, new lesions alone may not constitute progressive disease.

^¶^ confirmation scans after ≥4 weeks to confirm durable MR/PR/CR are always required. If confirmed, MR/PR/CR is backdated to the date of preliminary MR/PR/CR. If a patient is lost to follow-up (censored) before confirmation, preliminary CR/PR/MR is considered SD.

^*^ disregard new non-CE lesions unequivocally ascribable to post-RT.

^Δ^ either new measurable lesions or previously non-measurable lesions that became measurable and grew =5x=5 mm.

° for CE-tumors, only CE lesions should be considered.

^†^ in mixed tumors, the assessment should be performed in-parallel for both CE and non-CE components, then combined.

‡ corticosteroid dose increase alone does not define PD.

^§^ confirmation scans for PD can be waived: in CE tumors >3 months after RT completion not treated with agents highly associated with PsP; in the evaluation of non-CE progression in non-CE tumors or mixed tumors.

CE, contrast-enhancing; CR, complete response; MR, minor response; PD, progressive disease; PsP, pseudoprogression; PR, partial response; SD, stable disease.

Compared to the unidimensional measurement for BM in RANO-BM and iRANO, two-dimensional measurements (the sum of bi-perpendicular diameters) were used in both iRANO and RANO 2.0 for malignant and low-grade gliomas. Volumetric analysis is added in RANO 2.0.

RANO criteria have been used in different clinical studies, leading to variability in response assessments. Chen et al. found that RANO-HGG and iRANO had high concordance for assessing PD in patients within 6 months of ICIs initiation (142). Youssef et al. (143) showed that RANO-HGG and mRANO demonstrated similar correlations between progression-free survival and overall survival in GBM patients. However, iRANO criteria did not add significant benefit in GBM patients who received ICIs. Douri et al. (144) demonstrated that RANO-BM criteria unreliably identified clinically relevant tumor progression (TP) in BM after stereotactic radiosurgery.

It should be noted that in the case of immunotherapy, new lesions alone cannot constitute progressive disease in RANO-BM, iRANO, and RANO 2.0. Often immunotherapy-based treatments require the induction of immune cell infiltrates (e.g. CD8+ T cell lymphocytes) which takes time to mount an effective immune response against the tumor. Thus, for patients with the appearance of new lesions within 6 months of the initiation of immunotherapy alone and without substantial neurological decline, the iRANO working committee recommends confirmation of radiographic progression by follow-up imaging in 3 months after initial radiographic evidence of progressive disease. RANO-BM group recommends progressive disease should not be solely defined by the appearance of new lesions but rather as a minimum 20% increase in the sum longest diameter of CNS target and new lesions, unequivocal progression of existing enhancing non-target CNS lesions, unequivocal progression of existing non-enhancing (T2/FLAIR) CNS lesions, or clinical decline related to the tumor. A short interval scan is advised if immune response-related radiographical changes are suspected. In contrast, RANO 2.0 defines any new measurable (≥10mm**×**10 mm) enhancing lesions as PD in the case where the baseline or best response demonstrates no measurable enhancing disease. If there is uncertainty regarding progression, close observation (e.g. re-evaluated at 4-week intervals) is recommended.

## 5 Atypical patterns of response to immunotherapy

Beyond the standard response and progression patterns in **Table 3**, atypical patterns after immunotherapy including pseudoprogression (PsP), hyperprogression, and dissociated response, are important for radiologists and oncology physicians, and summarized in **Table 4**. Due to the increasing growth of combination treatments of immunotherapy, radiosurgery, and Bevacizumab, these atypical patterns are becoming common. The radiographic phenomena of radiation necrosis (RN), abscopal response, and pseudoresponse are summarized in **Table 4** and are discussed in the following section.

**Table 4:** Atypical patterns of response to immunotherapy, and their clinical implications.

Atypical pattern	Definition and clinical implication
Pseudoprogression	-Transient enlargement of primary lesion(s) or appearance of new lesion(s) after immunotherapy, followed by stabilization or shrinkage -Early response to immunotherapy rather than true tumor progression -Close observation by short interval scan -Continue treatment benefit instead of cessation of immunotherapy
Hyperprogresssion	-Atypical flare-up of tumor growth kinetics induced by immunotherapy -With potential deleterious impact and may lead to premature death -Early identification should be performed -Treatment modification
Dissociated Response	-Mixed imaging responses in which the tumor sizes of some lesions increase and others decrease or stabilize after immunotherapy -To continue immunotherapy, combining with local treatment can be discussed

### 5.1 Pseudoprogression

PsP is an imaging phenomenon describing the initial and transient growth of primary lesion(s) or the appearance of new lesion(s) after chemoradiotherapy or immunotherapy treatment, followed by stabilization or shrinkage. PsP is typically observed in the first 12 weeks (about 3 months) after completion of radiochemotherapy for GBMs (occurring in up to 30%-40% of patients) (141). This phenomenon can be observed in several different cancers following immunotherapies, including solid tumors, lymphomas, and brain tumors. Thus, PsP is one of the primary factors driving updates of the RANO criteria over the past 14 years.

The overall incidence of PsP ranges from 6% -14.8% (3, 145), varying with the type of tumor treated with ICIs. One study noted PsP in 17.9% of pembrolizumab treated patients with reported progression who were re-imaged after continued therapy despite reported progression (146). The PsP has also been reported in oncolytic viruses (141), ADCs, and CAR-T cell therapies (3). The mechanism underlying PsP is due to infiltration of T cells into the tumor environment leading to increased tumor appearance rather than true proliferation of tumor cells. Unrecognized PsP may cause early treatment cessation in patients who would benefit from continued immunotherapy (3).

### 5.2 Radiation necrosis

Due to the increasing application of combination treatment of radiotherapy and immunotherapy, and some BM patients had SRS before immunotherapy treatment, the term “RN” should be recognized in the treatment response evaluation. RN is defined as the occurrence of necrotic brain tissue after radiation therapy and usually occurs between three months and one year after radiotherapy (54). RN occurs in approximately 20% of radiotherapy in GBM patients (54) and 5%-25% of patients with BM treated with SRS (147).

PsP and RN are two different types of therapeutic effects and require completely different clinical approaches. RN can be treated conservatively with steroids or surgical removal due to a space-occupying aspect, or antiangiogenic drugs to alleviate brain edema. A pseudoprogression is usually treated conservatively.

### 5.3 Hyperprogresssion

**Figure 4:**
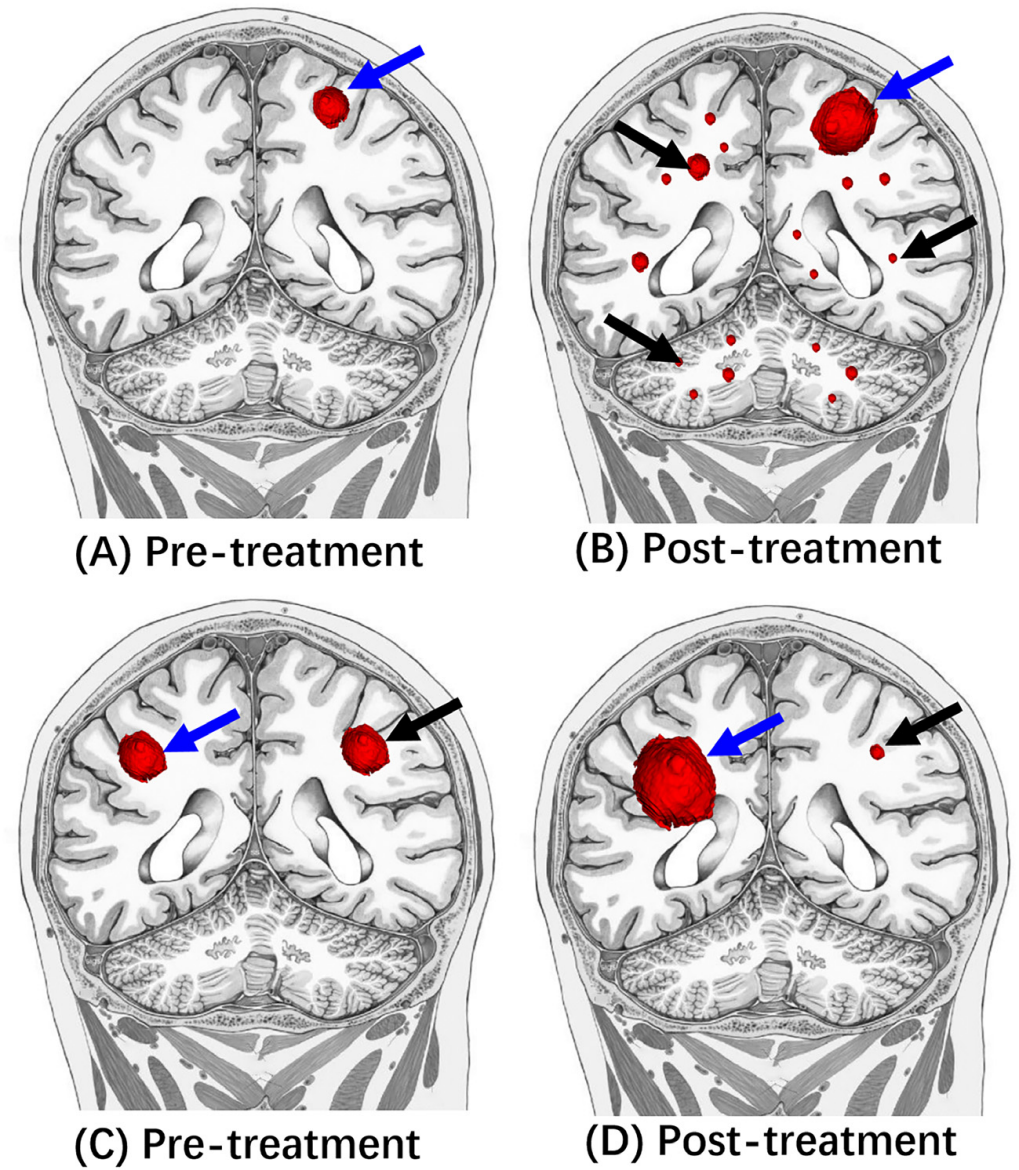
Illustrative diagrams of Hyperprogression and dissociated response. **(A, C)** Are pre-treatment MRI. Compared to **(A, B)** showed enlarged left frontal mass (blue arrow) and multiple new small masses (black arrow) in bilateral cerebral hemispheres and cerebellums after the immunotherapy treatment. Compared to the **(C, D)** showed the decreased size of left frontal mass (black arrow) after the immunotherapy treatment. In contrast, the right frontal mass (red arrow) increased the tumor size.

Hyperprogresssion is immunotherapy-induced acceleration of tumor growth leading to premature death (3, 145, 148, 149), **Figure 4**.

Various definitions of hyperprogression have been used in published studies. The pure radiological criteria define and analyze the variation of tumoral burden before and after initiation of immunotherapy, including at least three radiological assessments (one several weeks before baseline, one at baseline, and one after initiation of ICIs) (145).

Champiat et al. described “hyperprogressive disease” as a RECIST progression at the first evaluation and a twofold or greater increase in the tumor growth rate (TGR, the percentage increase in tumor volume per month) after starting PD-1/PD-L1 inhibitor therapy compared with the period before initiation of PD-1/PD-L1 inhibitor therapy (150).

Saada-Bouzid et al. evaluated tumor growth kinetics (TGK) based on the difference of the sum of the largest diameters of RECIST target lesions per unit of time between two evaluations. Hyperprogression was defined as TGK ratio (ratio of TGK after immunotherapy to pre-treatment TGK) ≥ 2 (145, 151).

Overall, the incidence of hyperprogression in solid tumors treated with immunotherapy (ICIs) differs widely in the literature, ranging from 4-29% (145, 149).

Statistically, worse overall survival was observed in patients experiencing hyperprogression than in patients experiencing standard progression. Therefore, hyperprogression has a potentially deleterious and fatal impact. Early identification of hyperprogression is crucial for patients treated with immunotherapy. Current immunotherapy regimens should be stopped promptly in case of suspected hyperprogression, and transition to other effective therapies must be performed (3, 145, 148, 149).

### 5.4 Dissociated response

Dissociated response, also known as a mixed response or disproportionate response, is a phenomenon demonstrating increased tumor size of some target lesions (which may qualify as progression) and decreased size or stability of additional target lesions following immunotherapy (3, 149), **Figure 4**.

Dissociated response may reflect the heterogeneity of tissue-specific tumor microenvironments and has been reported in less than 10% of patients (149).

Clinical management of dissociated response is still contested. Dercle et al. recommended continuing immunotherapy for durable clinical benefit despite RECIST1.1 progression (149). Kim et al. suggested combining immunotherapy with local treatment in the case of oligometastatic disease progression (3).

### 5.5 Abscopal response

In 1953, Mole first used the term ‘abscopal’ to report a phenomenon of out-of-field regression of unirradiated distant tumor sites following irradiation of a single tumor lesion (152). The abscopal response is an immune-mediated response to radiation by tumor cells located distant from the irradiated site. The mechanism may be related to released systemic tumor antigens (from local treatment), which activate the antitumor immune system, resulting in remote tumor regression outside of the radiation field (149). This once rare phenomenon may become more common in an era of increasing combination treatments of radiotherapy and immunotherapy (153).

### 5.6 Pseudo-response

Pseudo-response is a phenomenon of progression that has been reported in high-grade glioma patients treated with antiangiogenic agents and is characterized by decreased contrast enhancement and enlargement of the non-enhancing lesion on T2WI/T2-FLAIR (140). Pseudo-response indicates un-enhancing tumor infiltrative dissemination (154). The clinical use of combination therapy of antiangiogenic treatment and immunotherapy highlights the imaging challenges of treatment response assessment criteria for immunotherapy which traditionally emphasizes enhancing lesion(s) (138).

## 6 Advanced imaging techniques

Advances in imaging have played a significant role in improving the evaluation and outcomes of brain tumor immunotherapy, particularly in accurately identifying pseudoprogression, optimizing treatment decisions, and enhancing the ability to predict treatment response. Immunotherapy is often accompanied by complex imaging changes, making these advancements crucial for more precise assessments. One of the major challenges in post-immunotherapy treatment imaging is the assessment of treatment effects, (i.e. distinguishing RN, PsP from TP, detection and monitoring of immune-related adverse events(irAEs)) which guide optimal clinical decision-making.

It is usually difficult to distinguish between RN-PsP and TP, as they may have similar morphology, contrast enhancement, edema, and mass effect on conventional MR. Advanced imaging techniques including MR perfusion-weighted imaging (PWI), magnetic resonance spectroscopy (MRS), chemical exchange saturation transfer (CEST), and positron emission tomography (PET), have been widely used in treatment response assessment of brain tumors, and have been suggested in RANO 2.0 criteria (141). These technologies provide precise data on tumor metabolism, blood flow, and biochemical characteristics, enabling clinicians to detect the actual response to immunotherapy earlier and more accurately and improve the post-treatment differential diagnosis between RN-PsP and TP.

Furthermore, the integration of radiomics and artificial intelligence (AI) into modern imaging techniques has enhanced the level of personalization in treatment. By efficiently analyzing large-scale imaging data and extracting hidden biological features, AI provides precise treatment feedback, enabling the accurate identification of patients who are responsive to immunotherapy. This improves treatment outcomes and extends patient survival. This data-driven decision-making approach has already influenced clinical treatment pathways, making immunotherapy for brain tumor patients more targeted and effective.

### 6.1 MR PWI

Of all advanced imaging techniques, MR PWI is the most common clinical practice to discriminate RN-PsP from TP. MR PWI provides hemodynamic information regarding tumor angiogenesis in brain tumors. There are three MR PWI sequences, including dynamic susceptibility contrast (DSC), dynamic contrast-enhanced (DCE), and arterial spin labeling (ASL). Compared to DCE, which is less commonly used, DSC and ASL are major and validated perfusion imaging tools for the diagnosis and management of brain tumors. MR DSC-PWI estimates the relative cerebral blood volume (rCBV) based on T2* signal intensity changes from the first passage of paramagnetic contrast agents through the cerebrovascular system. ASL measures cerebral blood flow (CBF) noninvasively using endogenous tracers of magnetized blood instead of contrast agents.

A key pathology characteristic of TP and tumor recurrence in malignant brain tumors is angiogenesis, which results in increased rCBV. In contrast, inflammation and necrotic tissue damage lead to decreased rCBV in RN and PsP. TP has significantly higher rCBV, associated with increased metabolic activity and increased neovascularization, than RN and PsP. These differences help differentiate between RN-PsP and TP in the era of radiation and chemoradiation treatment.

Multiple studies have demonstrated that the mean and maximal rCBV (rCBV_max_) of TP in malignant gliomas were significantly higher than in PsP lesions (155–158). Anil et al. suggested the optimum standardized rCBV threshold of 1.64 for distinguishing tumor recurrence and post-treatment radiation effects (with a sensitivity of 84.48%, specificity of 84.97%, and accuracy of 84.73%). In contrast, the objective threshold of 1.75 obtains a sensitivity of 81.94%, specificity of 87.23%, and accuracy of 84.58% (157).

Previous studies also found that rCBV values of RN in BM were significantly lower than the rCBV values of TP and tumor recurrence (159–163). The recommended rCBV cut-off points in these studies ranged from 1.23 to 2.12. Mitsuya et al. analyzed 7 recurrent BM lesions and 21 RN lesions in 27 patients with BM undergoing SRS; they reported the best distinguishing cutoff was 2.1, with a sensitivity of 100% and a specificity of 95% (160). Umemura et al. assessed the ability of dynamic contrast-enhanced T1 MRI (DCE-MRI) perfusion to identify pseudoprogression in melanoma brain metastases, reporting an AUC of 0.75 for this diagnostic method (164).

There are few MR PWI studies evaluating the treatment response assessment in brain tumors following immunotherapy (165, 166), **Figures 2** and **4**. Cuccarini et al. analyzed 18 TP and 8 PsP lesions in 22 patients with newly diagnosed GBM treated with dendritic cell immunotherapy; they found that the difference of △rCBV_max_ could effectively differentiate tumor recurrence from PsP, with a sensitivity of 67% and specificity of 75% (p = 0.004), suggesting that the rCBV modifications over time might be more helpful to distinguish PsP from TP (167). In a retrospective study by Vrabec et al. (168), the authors reviewed 32 follow-up MRI examinations in 8 recurrent GBM patients treated with dendritic cell immunotherapy; they found the highest rCBV_max_ value (9.25 ± 2.68) was observed in the contrast-enhancing area of TP lesions, and the rCBV_max_ value was higher even at time points before definite progression (4.87 ± 1.61) compared to the rCBV_max_ value of stable lesions (1.22 ± 0.47). They suggested that the rCBV_max_ value was a potential radiological indicator to distinguish between immunotherapy-induced inflammatory response and recurrent GBM tumor growth. In a study that enrolled 79 examinations with DSC-PWI in 6 surgically/immunogene-treated GBM patients and two surgically treated GBM patients, Stenberg et al. showed that elevated rCBV, corresponding to the contrast-enhancing lesion, supports the diagnosis of recurrent GBM (169).

In a study conducted by Song et al. (170), they demonstrated that out of the 19 patients with recurrent GBM treated with ICIs, 12 were determined to have TP. In contrast, 7 had treatment responses after 6 months of ICI treatment. The authors found that pre-ICI and post-ICI treatment rCBV values of the TP group were higher (2.16 and 2.2 respectively) than the rCBV values of the treatment response group (pre-treatment, 1.67, p=0.25, and post-ICI treatment, 1.90, 0.89). However, it is interesting that the post-ICI treatment rCBV values of both the TP group and the treatment response group were increased compared to the pre-ICI treatment rCBV values. The authors thought that the absolute rCBV value or interval change in rCBV was not indicative of treatment response within 6 months. A possible reason for the absence of a significant difference in rCBV parameters between the TP group and the treatment response group was speculated to be related to the confounding effects of anti-angiogenesis treatment (165). In Song et al’s study, there were 5 patients (26.3%) treated with bevacizumab before the commencement of ICIs and remained on this therapy throughout the entire period of study follow-up. The bevacizumab can result in “pseudo-response” non-enhancing TP accompanied by decreased rCBV in malignant gliomas which may limit the generalizability of the findings.

### 6.2 MRS

MRS is a non-invasive molecular imaging technique that detects the local resonance frequencies of hydrogen nuclei in various biochemical compounds, enabling *in vivo* quantification of metabolites within tissues (54, 171). The major metabolites obtained in MRS include N-acetyl-aspartate (NAA), a neuronal viability marker, choline (Cho), indicative of cell membrane turnover, creatine (Cr), a stable internal standard, lactate, marking anaerobic metabolism, and myo-inositol, a marker of gliosis. These metabolites provide crucial insights into the biochemical environment of brain tumors and have been widely used in multiple studies and clinical practice (172).

MRS can differentiate between primary brain tumors and BM based on spectral patterns of metabolites (171). Primary brain tumors, such as GBMs, generally shows elevated Cho/Cr and Cho/NAA ratios due to increased cell membrane turnover and proliferation. BM is characterized by elevated Cho, lactate, and lipids, and the absence of NAA. The metabolite ratios of lipid/Cho and lactate/Cr can be used to differentiate RN-PsP from TP (171). A recent meta-analysis suggested that MRS had the highest diagnostic accuracy in distinguishing between treatment-related changes and tumor recurrence, with a sensitivity and specificity of 91% and 95% (172).

Furthermore, Floeth et al. found that the metabolic data of MRS may help to distinguish between tumor recurrence and PsP after local immunotherapy of GBM (173). However, MRS studies in the BM treated with immunotherapy are still limited.

### 6.3 CEST

CEST is another non-invasive advanced molecular imaging technique that focuses on the concentration and exchange rates of mobile proteins and peptides within brain tissue by monitoring the exchange of protons between mobile proteins or peptides and surrounding water molecules (174). For instance, amide proton transfer-chemical exchange saturation transfer (APT-CEST) can be used to measure protein concentrations, and glucoCEST can assess glucose metabolism; both processes are higher in brain tumors compared to normal brain tissue (99). CEST provides endogenous MRI contrast without the need for exogenously administered contrast agents. Thus, CEST is a valuable tool in the analysis of tumor microenvironment (174).

CEST shows great potential in glioma grading and monitoring treatment response (172).

In the context of post-immunotherapy treatment imaging, CEST imaging, especially APT-CEST, has shown significant promise. Studies have demonstrated its ability to distinguish between tumor recurrence and treatment-related changes such as PsP in patients with BM and gliomas (172). Due to increased protein and peptide content in viable tumor components, TP or tumor recurrence of gliomas presents a high signal on APT-weighted CEST images, while PsP shows iso- to mild hyperintensity (172). Ma et al. (175) applied amide proton transfer-weighted (APTW) MRI to distinguish pseudoprogression from true progression in 32 patients with malignant gliomas after chemoradiation. They reported an optimal cutoff APTW_mean_ of 2.42%, achieving a sensitivity of 85.0% and a specificity of 100%. CEST could accurately and promptly distinguish between PsP and TP in brain tumors after immunotherapy. Thus, CEST imaging greatly improves the comprehensive post-immunotherapy treatment assessment and clinical management in patients with brain tumors.

### 6.4 PET

PET is a nuclear medicine imaging technique that evaluates metabolic activity using radioactive tracers (147, 171). PET can distinguish between RN-PsP and TP based on metabolic differences. Specifically, TP and tumor recurrence lesions usually demonstrate active/increased metabolism. In contrast, RN and PsP have decreased/inactive decreased/tracer uptake (147, 171). Although the ^18^F-fluorodeoxyglucose (FDG) is the only FDA-approval tracer in the US, the effectiveness of FDG-PET in the treatment response assessment of brain tumors is limited, primarily due to underlying brain metabolism and spatial resolution (3, 171).

Various amino acid-based radiotracers, such as L-methyl-^11^C-methionine (MET), O-2-^18^F-fluoroethyl-L-tyrosine (FET), and 3,4-dihydroxy-6-^18^fluoro-L-phenylalanine (FDOPA), can effectively distinguish between tumor recurrence, PsP, and RN in malignant gliomas and BM (174). Previous studies have demonstrated the potential of PET/MRI in evaluating the therapeutic response of GBM, the potential benefits of FLT-PET/MRI for diagnosing melanoma BM and monitoring targeted immunotherapy treatments (176). In a study of immunotherapy with DC vaccination in GBM patients, ^18^F-FET PET imaging showed more accurate discernment than contrast-enhanced MRI initially (177). In addition, Joseph et al. speculated that the PET probe for deoxycytidine kinase (dCK) could be used to distinguish between immune inflammatory response and enhancement foci caused by other factors in contrast-enhanced MRI imaging (178). Galldiks et al. assessed ^18^F-FET PET for monitoring immune checkpoint and targeted therapy responses in 40 patients with melanoma and lung cancer BM, identifying a tumor-to-brain ratios threshold of 1.95 to distinguish relapse from treatment effects, with an accuracy of 85% (179).

Immuno-PET is an advanced imaging technique that shifts the focus from traditional tumor cell visualization to the comprehensive analysis of the tumor’s immune environment. This method employs radiotracers to target specific immune markers such as PD-L1, CD8, and various macrophage-associated biomarkers, providing valuable insights into the density, composition, and functional state of tumor-infiltrating leukocytes. Such imaging can predict the efficacy of immunotherapy and overall prognosis more accurately than traditional methods. This method offers a whole-tumor perspective that surpasses the limitations of single biopsy samples. In the future, the optimal strategy may shift from non-immune-specific imaging biomarkers to immune-specific biomarkers assessed with immuno-PET (180).

The performances of advanced imaging modalities in distinguishing treatment-related effects from true tumor progression are summarized in **Table 5**.

**Table 5:** Performance review of advanced imaging modalities in differentiating treatment-related effects with true tumor progression.

Advanced imaging techniques	Clinical Utility	SN	SP	AUC
MR PWI	DCE-MRI perfusion parameters could distinguish between pseudoprogression and progression in immunotherapy-treated melanoma brain metastases (164).	0.67	0.86	0.75
	Establish dual-echo DSC-MRI based fractional tumor burden mapping to differentiate recurrent tumor from treatment effects in patients with GBM (157).	0.84	0.85	0.94
	Perfusion weighted magnetic resonance imaging to distinguish the recurrence of metastatic brain tumors from radiation necrosis after stereotactic radiosurgery (160).	1.00	0.95	0.98
	The analysis of modifications over time in ADC and CBV can help differentiate PsP from TTP at onset during immunotherapy with dendritic cells in patients with glioblastoma (167).	0.67	0.75	0.81
MRS	MRS had the highest diagnostic accuracy in distinguishing between treatment-related changes and tumor recurrence in patients with high-grade glioma (172).	0.91	0.95	NA
CEST	APT-CEST helps accurately distinguish true tumor progression from pseudoprogression in glioma patients undergoing standard treatment (175).	0.8*, 0.95^#^	1.0*, 0.92^#^	0.98*, 0.97^#^
PET	Monitoring Recurrence and Therapeutic Response Using ^18^F-FET PET in Patients with Melanoma and Lung Cancer Brain Metastases Undergoing Immunotherapy and Targeted Therapy (181).	0.70*, 0.80^#^	0.94*, 0.83^#^	0.85*, 0.78^#^
Radiomics and AI	A computed tomography (CT)-based radiomics model capable of precisely predicting hyperprogression and pseudoprogression (PP) in patients with non-small cell lung cancer (NSCLC) treated with immunotherapy (182).	0.92*, 0.83^#^	0.86*, 0.92^#^	0.95*, 0.97^#^
	MRI-derived radiomics assessing tumor-infiltrating macrophages enable prediction of immune-phenotype, immunotherapy response and survival in glioma (183).	NA	NA	0.71 (1 Year), 0.73 (2 Year), 0.68 (3 Year)
	Using a multiparametric magnetic resonance imaging-based radiomics model to distinguish glioma recurrence from pseudoprogression (184).	0.87	0.94	0.96
	Developed and validated a radiomic model called CRN, which utilizes T1CE images to evaluate the intracranial reaction to immunotherapy in NSCLC patients with BMs (185).	0.77*, 0.79^#^	0.85*, 0.79^#^	0.89*, 0.83^#^

SN, Sensitivity; SP, Specificity; AUC, Area Under the Curve; NA, Not Available; PWI, perfusion-weighted imaging; MRS, magnetic resonance spectroscopy; CEST, chemical exchange saturation transfer; PET, positron emission tomography; AI, artificial intelligence; NSCLC, non-small cell lung cancer; ADC, Apparent Diffusion Coefficient; CBV, cerebral blood volume. * indicates the result of the training set, # indicates the result of the validation set.

### 6.5 Radiomics and AI

Radiomics is a rapidly developing field of research focused on the extraction of quantitative features from medical images, thus converting these digital images into minable, high-dimensional data, which offer unique biological information that can enhance our understanding of disease processes and provide clinical decision support (186). Artificial intelligence (AI) essentially simulates human cognitive and decision-making abilities through machine learning and deep learning algorithms. It relies on continuously adapting and learning from large-scale data to optimize models, thereby enhancing their capability to handle complex tasks (187).

The combination of artificial intelligence methods and radiomics has been widely applied in tumor diagnosis, classifications (e.g., primary and secondary tumors), discriminations between treatment effects (pseudoprogression, radiation necrosis) and true progression, survival prediction, inflammation, and identification of tumor biomarkers (188). These models can predict the efficacy of ICI through imaging features, accurately identifying patients who may benefit from ICI therapy, thus advancing the development of personalized immunotherapy. In addition, AI can discover new immunotherapy targets, aiding in the design of targeted treatment strategies. Through deep learning techniques, AI has revealed mechanisms of resistance and patterns of recurrence in tumor immunotherapy by exploring multi-omics data, helping to understand the molecular mechanisms of resistance.

Currently, the application of AI algorithms and radiomics in solid tumors beyond the CNS has demonstrated great potential in predicting the efficacy of immunotherapy (189). Sinha and colleagues have summarized that AI or machine-learning (ML) models can accurately predict immune therapy responses, progression-free survival, and overall survival in NSCLC patients (189). Li and colleagues used radiomics to develop predictive models for pseudoprogression and high progression in NSCLC patients receiving ICI therapy. The AUC values for the training and validation sets were 0.95 and 0.88, respectively (182). Additionally, Shu and colleagues (190) emphasized that radiomics helps predict immune checkpoint inhibitor-related pneumonitis (ICIP) in NSCLC patients, providing new methods and insights for the early diagnosis and management of severe adverse reactions caused by immunotherapy. Furthermore, Prelaj and colleagues (191) demonstrated that AI-based approaches, combined with radiomics and pathology, have expanded the discovery of new biomarkers for immunotherapy.

Compared to research on solid tumors in other body systems,the studies on the application of AI are relatively fewer in brain tumors. In particular, there are still many unmet needs in predicting the response to immunotherapy. The timing of pseudoprogressive changes in BM patients treated with ICIs has not been fully explored, reports on hyperprogression after initiation of ICI monotherapy remain scarce (181). However, preliminary studies have shown that AI algorithms exhibit potential for predicting survival outcomes and treatment responses after immunotherapy (99, 192).

The use of AI in glioblastoma patients primarily focuses on diagnosis and treatment planning (193). Studies have found that MRI-derived radiomics assessing tumor-infiltrating macrophages enable prediction of immune-phenotype, immunotherapy response and survival in glioma (183). The immunoradiomics model they developed could allow for non-invasive assessment of the absolute density of tumor-associated macrophages from MRI images in HGG patients, and effectively predicted the patients’ survival outcomes (183). Additionally, Fu and colleagues (184) developed a radiomics-based model to differentiate between glioma recurrence and pseudoprogression, with the best predictive model achieving an AUC of 0.96 (**Table 5**). AI models have also shown potential in predicting immune-related adverse events (irAEs) and patient survival, helping to mitigate the risks associated with more potent immunotherapies (180). Sinigaglia and colleagues found that ML algorithms and AI signatures were trained to predict overall survival in patients with solid tumors treated with ICM based on pretreatment-imaging biomarkers. These biomarkers, are identified as predictors of poorer outcomes (193).

In brain metastases (BMs), AI and radiomics are not only used to identify the type and mutation status of the primary tumor but are also increasingly applied to assess tumor response after immunotherapy (99). Xu and colleagues (185) successfully used AI and radiomics to predict progression-free survival and overall survival in brain metastasis patients receiving ICIs. Their research suggests that pre-treatment radiomic features may help in the early prediction of treatment benefits. Galldiks and colleagues found that imaging parameters obtained through amino acid PET, including radiomic features, have significant value in the diagnosis of recurrence and evaluation of treatment efficacy in brain metastasis patients (194). Xu and colleagues’ research further indicated that radiomic biomarkers from pre-treatment MRI images can effectively predict intracranial response (185). However, studies on the use of neuroimaging biomarkers for non-invasive detection remain relatively limited.

AI and radiomics have demonstrated great potential in neuro-oncology, providing advanced tools for diagnosis, treatment monitoring, and prognostic evaluation. By characterizing tumor biology through medical imaging, radiomics combined with AI-derived biomarkers offers hope for personalized medicine in the era of immunotherapy. Compared to traditional imaging techniques, AI demonstrates higher sensitivity and accuracy in assessing immune responses in neuro-oncology. Using radiomics, AI extracts complex micro-level features and can detect early immune responses before visible changes in the tumor (195). AI reduces human error and improves the consistency and standardization of diagnostic and treatment decisions. Additionally, it tailors predictions based on individual patient characteristics (e.g., imaging, genetics, clinical data), providing strong support for personalized and precise treatments (196).

Despite the rapid development of ML technologies in recent years, their broad application in neuro-oncological radiology still faces numerous challenges. Firstly, AI models rely on large datasets for training, but the limited sample sizes in neuro-oncology immune therapy studies reduce their effectiveness. Secondly, inconsistencies in imaging data from different devices lead to unstable model performance across datasets, limiting widespread AI adoption. Additionally, poor model interpretability and a lack of transparency in decision-making reduce clinical trust (197). Lastly, most AI models rely on static imaging data, while immune therapy effects evolve over time, limiting the models’ ability to predict dynamic responses (198).

In summary, despite the significant potential of AI in predicting immune therapy responses and outcomes, future research must address issues such as data sample limitations, cross-device compatibility, and model interpretability. Resolving these challenges is essential for the broader clinical application of AI and to enhance precision in personalized medicine.

## 7 Immune-related CNS adverse events and the spectrum of imaging manifestations

Immunotherapy can produce a spectrum of toxicity involving organs due to autoimmune effects from misdirected stimulation of the immune system, termed irAEs. The irAEs include pneumonitis, colitis, hepatitis, pancreatitis, thyroiditis, hypophysitis, synovitis, arthritis, and even sarcoid-like granulomatosis and lymphadenopathy. irAEs have been an increasingly recognized concept in cancer imaging over the past few years. The mechanism of irAEs is presumed to be autoimmune effects resulting from misdirected stimulation of the immune system during immunotherapy. In addition to the autoimmune effects, the T-cell inflammatory responses can produce swelling in the targeted tumor microenvironments that resemble tumor growth, as well as disease spread into susceptible healthy tissues. Thus, it can be difficult to distinguish between response and progression during early treatment. The accurate recognition of pseudoprogression and early diagnosis of irAEs is essential to the clinical management of immunotherapies (148, 149).

Due to the rapidly increasing uses of immunotherapy in clinical neuro-oncology practices, there are increasing demands for neuroradiologists and clinicians to be familiar with imaging manifestations of irAEs in CNS. The irAEs in CNS include encephalopathy, ICIs-induced autoimmune or limbic encephalitis, hypophysitis, posterior reversible encephalopathy syndrome (PRES), multiple sclerosis (MS), aseptic meningitis, transverse myelitis, necrotizing myelopathy, and vasculitis (199).

**Figure 5:**
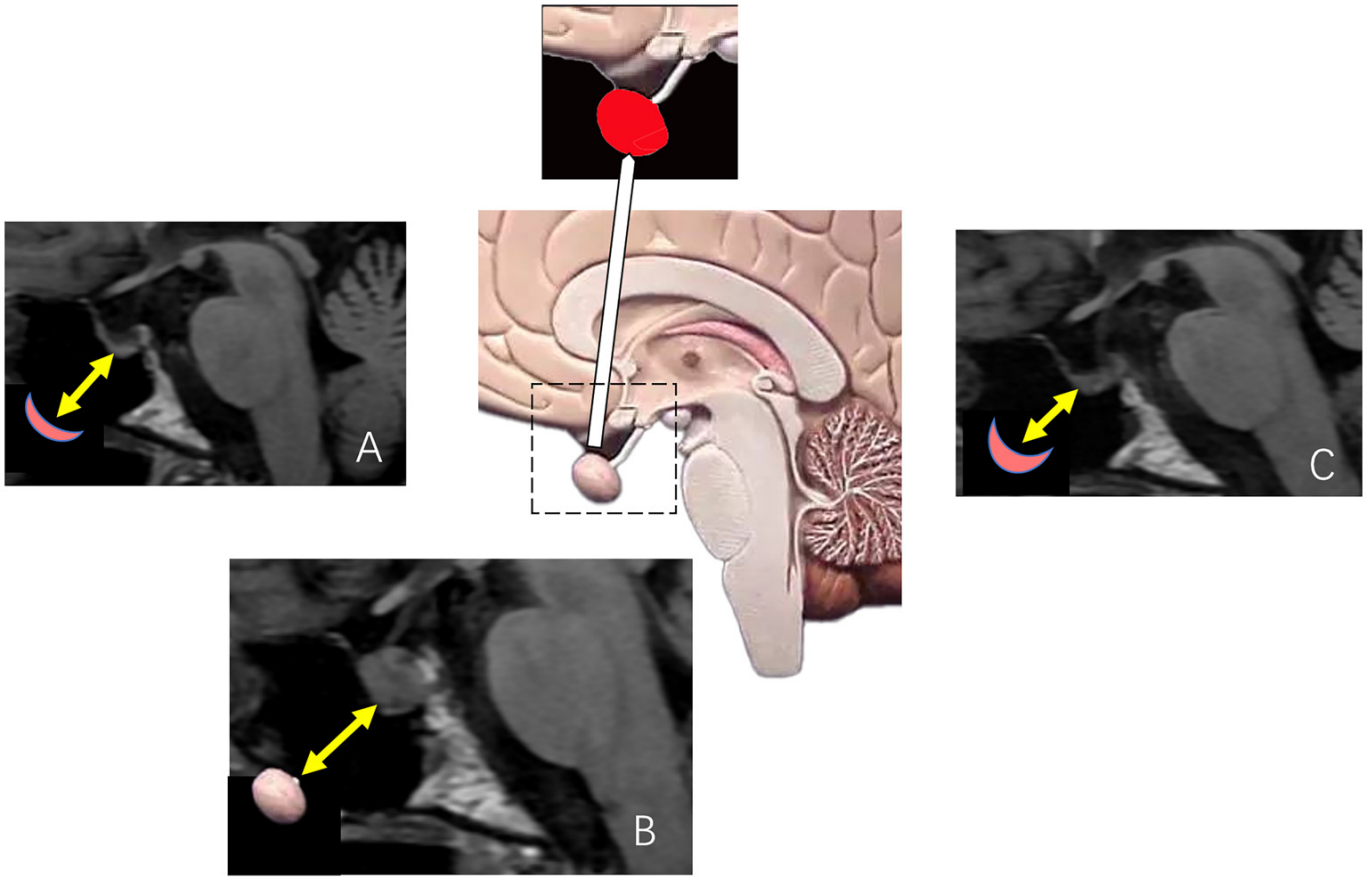
The diagram of the pituitary gland and an illustrative case of hypophysitis induced by ipilimumab treatment. **(A–C)** Are sagittal T1-FLAIR images of a patient with melanoma treated with ipilimumab. **(A)** Is a pre-immunotherapy MRI showing the unremarkable size of the pituitary gland (yellow arrow). During the ipilimumab treatment, the patient presented a sudden onset of intractable headache and nausea. **(B)** Demonstrated significant and heterogeneous enlargement of the pituitary gland (yellow arrow). Follow-up MRI after 6 weeks, **(C)** showed resolution of pituitary enlargement (yellow arrow).

Hypophysitis is the most common pattern of the CNS irAEs, this immune-related endocrinopathy can be observed in approximately 10%–13% of patients with melanoma treated with ipilimumab, **Figure 5**.

The clinical symptoms of immune-related hypophysitis are commonly headache, fatigue, weakness, and/or rarely visual defects (likely because the enlargement of the pituitary gland is usually mild). Hypophysitis diagnosis is generally based on the development of new hypopituitarism and pituitary enlargement at imaging after initiation of immunotherapy without an alternative etiology. Enlarged pituitary glands may demonstrate homogeneous or heterogeneous enhancement at MRI and thickening of the pituitary stalk may also be present. Resolution of pituitary enlargement after high-dose corticosteroid administration treatment can be observed at follow-up MRI (148, 149, 199).

Kurokawa et al. suggested an additional MRI feature of ICI-induced hypophysitis as hypo-enhancing geographical areas of low T2 signal in the anterior pituitary without blooming artifact. This pattern typically reflects fibrosis rather than necrosis or hemorrhage (200).

This specific MRI characteristic provides a way to distinguish ICI-induced hypophysitis from other types of hypophysitis or neoplasms (148, 149, 199, 200).

Autoimmune encephalitis induced by ICIs lacks distinctive MRI patterns, MRI may reveal T2/FLAIR hyperintensities affecting white and deep gray matter, including the lentiform nuclei and external capsule/claustrum (201). Limbic encephalitis has also been reported with characteristic MRI appearance of symmetrical inflammatory changes in bilateral mesial temporal lobes. The variability in MRI findings, coupled with often negative results in auto-immune antibody panels, contributes to diagnostic challenges (202).

## 8 Challenges and perspective

Despite many promising neuro-imaging studies in brain tumors treated with immunotherapy, there are still multiple challenges in this field, especially for the successful application of advanced imaging techniques.

### 8.1 Physical limitations: size and location

1a. Due to the inherent limitation of imaging resolution, advanced imaging techniques can’t be used for small lesions. Additionally, these imaging techniques are often limited for cystic gliomas or cystic BMs, where the thin wall may result in an undetectable rCBV (54, 171, 174).

1b. The proximity of brain tumor lesions to the cerebral cortex and skull limits the application of advanced imaging techniques, primarily due to artifactual signals.

### 8.2 Pathological limitations

**Figure 6:**
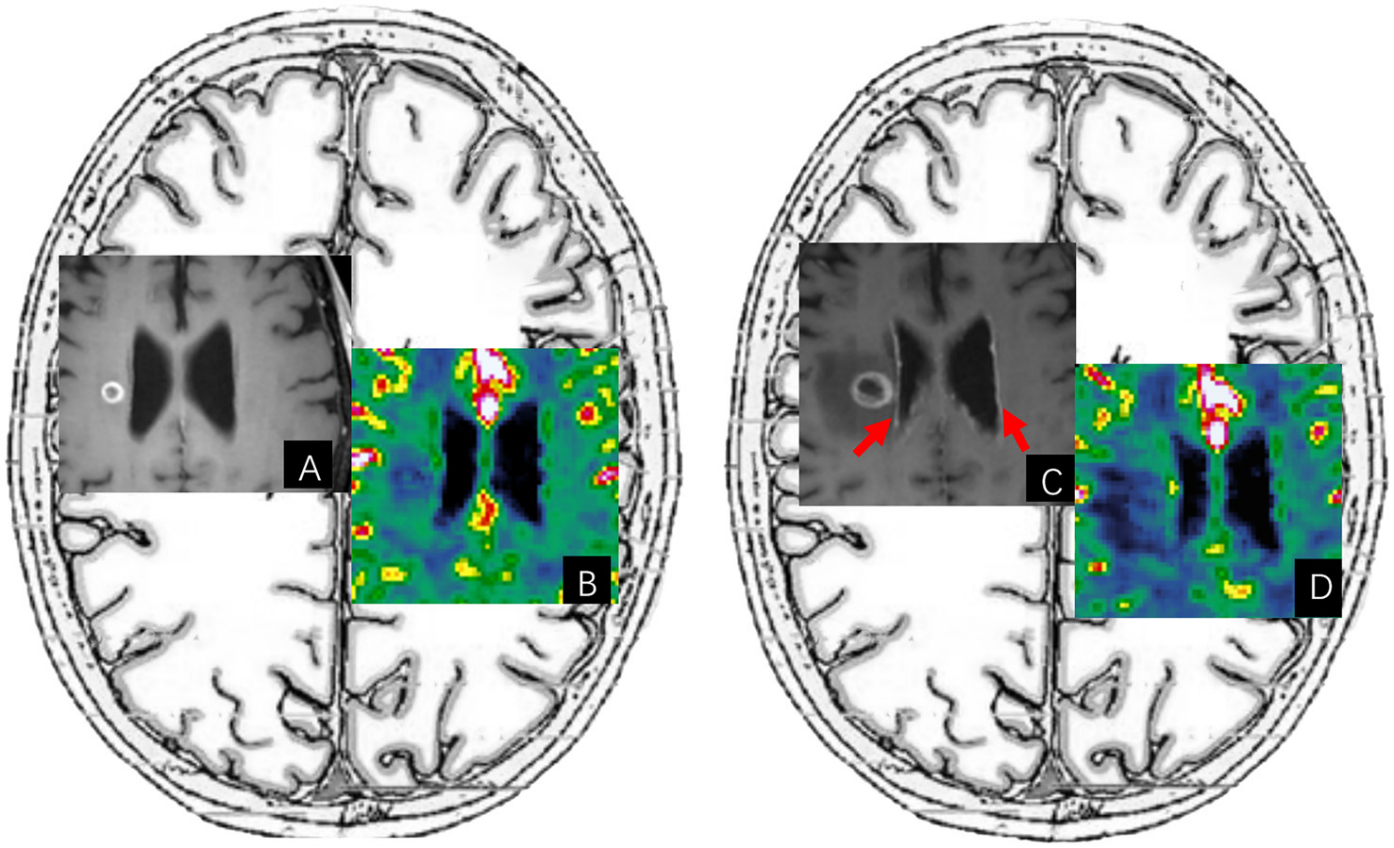
An illustrative case of diffuse subependymal progression after immunotherapy. **(A, B)** Are pre-immunotherapy post-contrast T1-FLAIR images and CBV maps of a patient with BM from renal cell carcinoma. After immunotherapy treatment, **(C)** of the post-contrast T1-FLAIR image showed diffuse subependymal enhancement (red arrow) in the lateral ventricles, accompanied by an enlarged ring-enhancing lesion and increased surrounding edema. These foci of subependymal enhancement were too small to be evaluated on the CBV map of **(D)**.

2a. A leptomeningeal lesion, especially leptomeningeal metastasis, is an important TP pattern in malignant brain tumors. However, the evaluation of a solitary leptomeningeal lesion and/or ependymal lesion (**Figure 6**) is often limited by advanced imaging techniques due to their location and they were excluded from the treatment response assessment of the RANO criteria.

2b. RN or PsP in the acute phase can present elevated rCBV which can create diagnostic uncertainty (171).

2c. Hemorrhage is a common pathologic feature, especially in post-treatment brain tumors. The MR PWI is limited by magnetic susceptibility artifacts due to petechial hemorrhage or melanin in melanomas.

2d. It has been noted that a large overlap of CBV_max_ values exists between the RN-PsP group and the TP group, leading to image interpretation challenges. This effect may be explained by pathologic characteristics including tumor heterogeneity and the similarity between microvascular density in active tumors and hyperplastic dilated blood vessels in post-radiation changes (54).

2e. Biopsy with histopathologic evaluation remains the gold standard to differentiate RN-PsP from TP. However, stereotactic biopsies can introduce sampling bias (147). In addition, it is common for the co-existence of RN and some active tumor/tumor or tumor recurrence in the tissue samples RN, the percentage is variable and possibly depends on the time of evolution (171).

2f. Bevacizumab, an antiangiogenic agent, inhibits vascular endothelial growth factor and thereby decreases endothelial cell proliferation and new blood vessel formation. After the administration of Bevacizumab, a rapid decrease in contrast enhancement and edema can be observed transiently (54). Bevacizumab can cause “tumor vasculature normalization” which subsequently leads to a decrease in perfusion within the tumor. Thus, combining immunotherapy and antiangiogenic treatment in malignant brain tumors may result in confounding rCBV interpretation, especially in the atypical non-enhancing TP pattern of “pseudoresponse”.

Despite the above imaging challenges, there is an urgent need for better imaging concepts to adapt to the novel patterns of immunotherapy response, including hyperprogression, PsP, and irAEs. Furthermore, the identification of risk factors and the establishment of comprehensive predictive models could help clinicians evaluate the benefit-risk ratio before starting immunotherapy in the future.

## 9 Conclusion

MRI plays an important role in the assessment of immunotherapy related treatment response, progression of brain tumors, and detection/monitoring for irAEs. The post-immunotherapy treatment imaging changes in brain tumors are more complicated. Novel imaging advancements should be implemented for improved post-immunotherapy treatment assessment in brain tumors, better personalized medicine, and improved management of such primary and metastatic brain malignancies patients.

## Glossary

CNS: central nervous system
FDA: food and drug administration
PD-1: programmed cell death protein 1
PD-L1: programmed death ligand-1
CTLA-4: cytotoxic T-lymphocyte-associated protein 4
ICI: Immune checkpoint inhibitor
NSCLC: non-small cell lung cancer
RCC: renal cell carcinoma
TNBC: triple-negative breast cancer
TIM-3: T cell immunoglobulin and mucin domain-containing protein 3
HCC: hepatocellular carcinoma
TIGIT: T cell immunoreceptor with Ig and immunoreceptor tyrosine‐based inhibition motif domains
SCLC: small cell lung cancer
NK: natural killer
HPV: human papillomavirus
HBV: hepatitis B virus
ACT: Adoptive Cell Therapy
TIL: Tumor-Infiltrating Lymphocytes
TCR: T-cell receptors
CAR-T: Chimeric Antigen Receptor T cells
ALL: acute lymphoblastic leukemia
IL-2: interleukin-2
IL-15: interleukin-15
IL-21: interleukin-21
TNF: Tumor necrosis factor
BBB: blood-brain barrier
GBM: Glioblastoma multiforme
BM: brain metastases
T-VEC: Talimogene laherparepvec
CAR-NK: Chimeric Antigen Receptor-Natural Killer
PCNSL: Primary central nervous system lymphoma
RANO: Response Assessment in Neuro-Oncology
RANO-HGG: response criteria for high-grade gliomas
RANO-LGG: response criteria for low-grade gliomas
RANO-BM: RANO Criteria for brain metastases
mRANO: Modified RANO Criteria
CR: complete response
PR: partial response
SD: stable disease
PD: progressive disease
TGR: tumor growth rate
TGK: tumor growth kinetics
RN: radiation necrosis
PsP: Pseudoprogression
TP: tumor progression
PWI: perfusion-weighted imaging
MRS: magnetic resonance spectroscopy
CEST: chemical exchange saturation transfer
PET: positron emission tomography
DSC: dynamic susceptibility contrast
DCE: dynamic contrast-enhanced
ASL: Arterial spin labeling
rCBV: relative cerebral blood volume
CBF: cerebral blood flow
irAEs: immune-related adverse events immune-related adverse events
NAA: N-acetyl-aspartate
Cho: choline
Cr: creatine
APT-CEST: amide proton transfer-chemical exchange saturation transfer
FDG: ^18^F-fluorodeoxyglucose
MET: L-methyl-^11^C methionine
FET: O-2-^18^F-fluoroethyl-L-tyrosine
FDOPA: 3,4-dihydroxy-6-^18^fluoro-L-phenylalanine
AI: artificial intelligence
ML: machine learning
MS: multiple sclerosis
PRES: posterior reversible encephalopathy syndrome
